# Cyanobacteria as cell factories for the photosynthetic production of sucrose

**DOI:** 10.3389/fmicb.2023.1126032

**Published:** 2023-02-14

**Authors:** María Santos-Merino, Lisa Yun, Daniel C. Ducat

**Affiliations:** ^1^MSU-DOE Plant Research Laboratory, Michigan State University, East Lansing, MI, United States; ^2^Department of Biochemistry and Molecular Biology, Michigan State University, East Lansing, MI, United States

**Keywords:** cyanobacteria, sucrose metabolism, carbohydrate feedstocks, osmoprotection, co-cultures

## Abstract

Biofuels and other biologically manufactured sustainable goods are growing in popularity and demand. Carbohydrate feedstocks required for industrial fermentation processes have traditionally been supplied by plant biomass, but the large quantities required to produce replacement commodity products may prevent the long-term feasibility of this approach without alternative strategies to produce sugar feedstocks. Cyanobacteria are under consideration as potential candidates for sustainable production of carbohydrate feedstocks, with potentially lower land and water requirements relative to plants. Several cyanobacterial strains have been genetically engineered to export significant quantities of sugars, especially sucrose. Sucrose is not only naturally synthesized and accumulated by cyanobacteria as a compatible solute to tolerate high salt environments, but also an easily fermentable disaccharide used by many heterotrophic bacteria as a carbon source. In this review, we provide a comprehensive summary of the current knowledge of the endogenous cyanobacterial sucrose synthesis and degradation pathways. We also summarize genetic modifications that have been found to increase sucrose production and secretion. Finally, we consider the current state of synthetic microbial consortia that rely on sugar-secreting cyanobacterial strains, which are co-cultivated alongside heterotrophic microbes able to directly convert the sugars into higher-value compounds (e.g., polyhydroxybutyrates, 3-hydroxypropionic acid, or dyes) in a single-pot reaction. We summarize recent advances reported in such cyanobacteria/heterotroph co-cultivation strategies and provide a perspective on future developments that are likely required to realize their bioindustrial potential.

## 1. Introduction

Product generation through heterotrophic microbial fermentation has been successfully used as an alternative approach to classical chemical processes using petroleum-based feedstocks (Blombach et al., 2022). However, bioindustrial chemical production by bacterial fermentation is still not economically competitive for many commodity products due in part to the high costs associated to the carbon substrates used for these organisms (Lee et al., 2022). Extensive research efforts have been expended to identify new plant species or to improve biomass processing technologies and increase the yield of fermentable sugars from plant feedstocks (e.g., improving carbohydrate recovery from cellulosic materials; Sun et al., 2022) and to overcome other land-use problems of plant-based feedstocks (Das and Gundimeda, 2022). Thus, there is an increased interest on the search for alternative, economical and environmentally sustainable sources as carbohydrate feedstocks.

Cyanobacteria and microalgae have attracted more attention in the last few years as an alternative supply for carbohydrates to support industrial fermentative processes (Hays and Ducat, 2015; Santos-Merino et al., 2019). In comparison with plants, cyanobacteria and algae can tolerate many water supplies that are unsuitable for agriculture (Santos-Merino et al., 2019; Catone et al., 2021), reducing their competition with food crops for the limited supply of arable land and freshwater. Microalgae and cyanobacteria are generally easier to manipulate genetically, have rapid division times, and can achieve higher efficiencies of solar energy capture and conversion (Santos-Merino et al., 2019). Relative to microalgae that tend to store excess carbon in the form of lipids or starch (Scott et al., 2010), cyanobacteria normally accumulate carbon reserves in polysaccharides and frequently sucrose as a compatible solute (osmolyte) in high-salt environments or under other abiotic stress (Klähn and Hagemann, 2011; Kirsch et al., 2019; Sanz Smachetti et al., 2020). Sucrose metabolism and its regulation has been amply studied in cyanobacteria (Kolman et al., 2015; Kirsch et al., 2019), while the activity and regulation of sucrose metabolism factors has received less attention in microalgae (Radakovits et al., 2010; Hagemann, 2016). The increasing knowledge on the synthesis and regulation of sucrose not only improves our understanding of these pathways but will also be useful for genetically engineering them for future biotechnological applications.

A number of cyanobacterial species have been effectively engineered to produce and secrete large amounts of sucrose by taking advantage of cyanobacterial sucrose biosynthesis pathways and heterologous co-expression of sucrose permease (CscB, Ducat et al., 2012; Du et al., 2013; Abramson et al., 2016; Kirsch et al., 2018; Lin P. C. et al., 2020) to export sucrose from the cell. In addition to batch cultures, there are increasing examples of real-time conversion of the carbohydrate feedstock through the direct co-culture of microbial partner strains that metabolize the secreted bacterial sucrose to higher-value products (Smith and Francis, 2016; Hays et al., 2017; Löwe et al., 2017; Weiss et al., 2017; Fedeson et al., 2020; Hobmeier et al., 2020; Zhang et al., 2020; Ma et al., 2022; Kratzl et al., 2023), potentially bypassing the costly processes of purifying and concentrating sucrose (Radakovits et al., 2010). However, to further use these synthetic light-driven microbial consortia in industrial applications, a number of challenges need to be overcome, such as long-term production stability, vulnerability to invasion by opportunistic microbial or viral contaminants, and imbalances in attributes of consortia that can contribute to inefficiencies (Hays and Ducat, 2015; Gao et al., 2022).

This review focuses on the current knowledge of the sucrose synthesis and degradation pathways in cyanobacteria as well as the list of genetic modifications in sucrose metabolic pathways that have been found to increase the production and secretion of this sugar. While other sugars can be produced phototrophically from cyanobacteria (e.g., glucose, fructose, or polysaccharides; Niederholtmeyer et al., 2010; Arias et al., 2021), sucrose has been the highest yielding carbohydrate reported and is the main focus of this review. We highlight some unresolved questions for additional study on fundamental cyanobacterial sucrose metabolism and the utilization of these pathways for bioproduction. Finally, we examine the current state of synthetic microbial consortia that capitalize upon the carbon fixation that photoautotrophs like cyanobacteria are uniquely able to provide.

## 2. Cyanobacterial sucrose metabolism

### 2.1. Sucrose biosynthesis pathway

**Figure 1:**
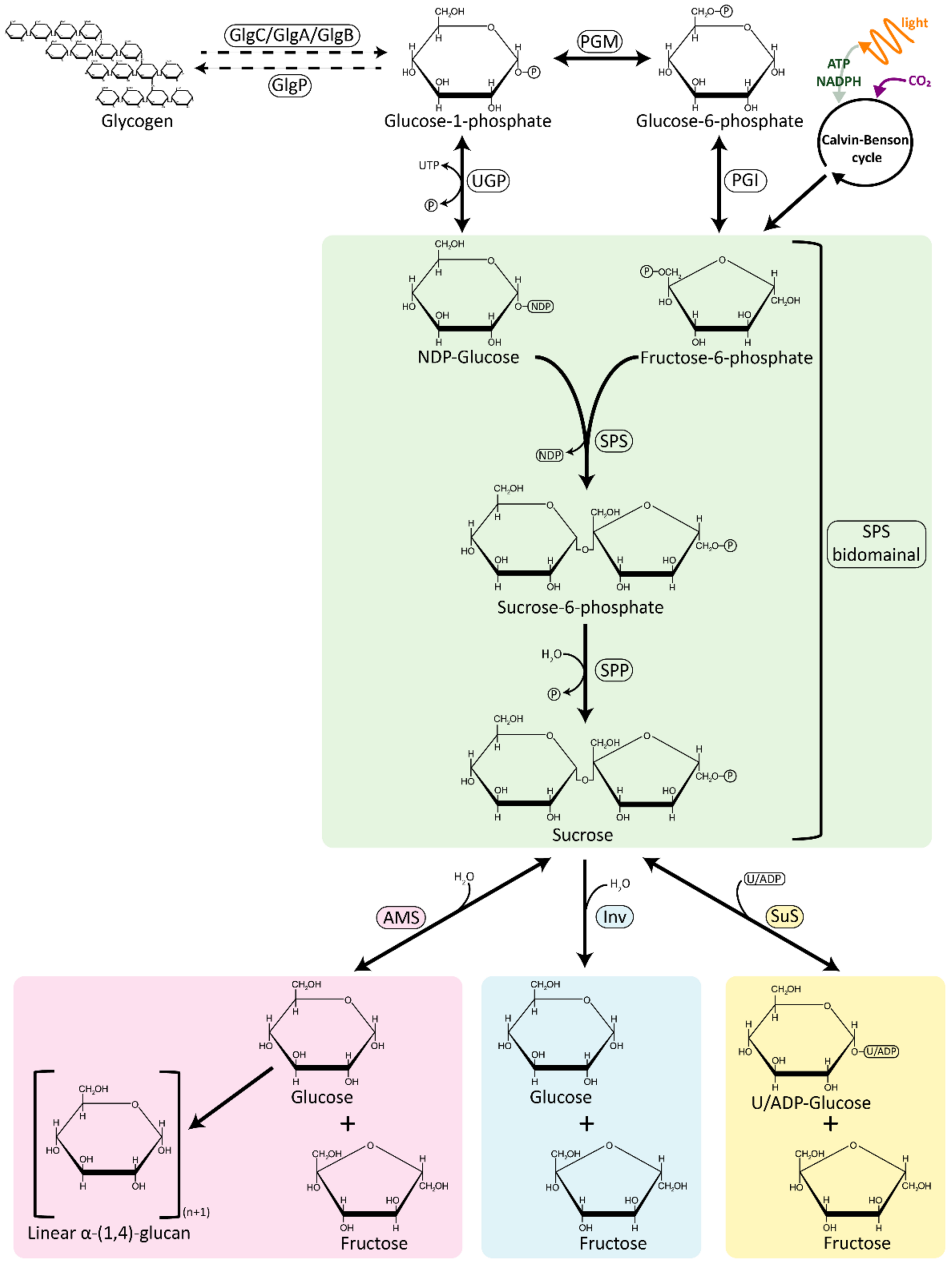
An overview of sucrose synthesis and degradation pathways in cyanobacteria. The sucrose synthesis pathway is represented in green; the degradation pathways are represented in pink, blue, and yellow. AMS, amylosucrase; GlgA, glycogen synthase; GlgB, glycogen branching enzyme; GlgC, ADP-glucose pyrophosphorylase; GlgP, glycogen phosphorylase; INV, invertase; PGI, phosphoglucose isomerase; PGM, phosphoglucomutase; SPP, sucrose phosphate phosphatase; SPS, sucrose phosphate synthase; SuS, sucrose synthase; UGP, UDP-glucose pyrophosphorylase.

Sucrose is a disaccharide [α-d-glucopyranosyl (1 → 2) β-d-fructofuranoside], whose synthesis pathway appears to be nearly universal among cyanobacteria, as predicted by the presence of sucrose synthesis genes in most of the known genome sequences available so far (Kolman et al., 2015; Kirsch et al., 2019; Supplementary Table S1). Sucrose synthesis lies close to the core of central carbon metabolism, with substrates directly derived from the Calvin-Benson-Bassham (CBB) cycle and its immediate downstream products (Figure 1). The light reactions of photosynthesis generate NADPH and ATP, which are used in the CBB to fix CO_2_ and yield glyceraldehyde-3-phosphate (GAP). GAP can be interchangeably converted to dihydroxyacetone phosphate (DHAP), and the condensation of GAP and DHAP through the activity of the enzyme fructose 1,6-bisphosphate aldolase (FBA), leads to the formation of fructose 1,6-bisphosphate (FBP). FBP is then further transformed into other hexose phosphates, such as fructose 6-phophate (F6P) and glucose 6-phosphate (G6P). G6P can be used to form nucleotide sugars such as uridine diphosphate glucose (UDP-Gluc).

Sucrose is most commonly synthesized from these CBB products in a two-step reaction by the sequential activity of two enzymes, sucrose phosphate synthase (SPS) and sucrose phosphate phosphatase (SPP; Figure 1). NDP-Gluc is combined with F6P to form sucrose 6-phosphate (S6P) in a reaction catalyzed by SPS. S6P is then dephosphorylated by SPP to sucrose, concluding the sucrose biosynthesis pathway. The rapid irreversible hydrolysis of S6P by a specific and high-activity SPP drives the reversible reaction catalyzed by SPS towards the direction of sucrose synthesis, even at low substrate concentrations (Lunn and ap Rees, 1990). An alternative route for sucrose synthesis is catalyzed by the enzyme sucrose synthase (SuS), which binds UDP/ADP-Gluc with fructose to produce sucrose (Porchia et al., 1999; Figure 1). While SuS is able to catalyze the synthesis of sucrose, cellular energetics are such that SuS is thought to be solely involved in sucrose cleavage *in vivo* (Curatti et al., 2002). SuS is ubiquitous across plant species, in contrast with cyanobacteria, where its occurrence is not widespread (Salerno and Curatti, 2003).

#### 2.1.1. Sucrose phosphate synthase

SPS catalyzes the first step in the pathway of sucrose synthesis by transferring a glycosyl group from an activated donor sugar, such as UDP-Gluc, to a sugar acceptor F6P, resulting in the formation of UDP and S6P (Figure 1). SPS (EC 2.4.1.14) is a UDP–glucose: d-fructose-6-phosphate 2-α-d-glucosyltransferase belonging to the GT-B (glucosyltransferase fold B) type glucosyltransferase family and its secondary structure consists of two distinct Rossmann-fold domains (super-secondary structures composed of consecutive alternating β-strands and α-helices that form a layer of β-sheet with one/two layer/s of α-helices) - a sugar acceptor domain (N-terminal “A-domain”) and a sugar donor domain (C-terminal “B-domain”; Chua et al., 2008; Lairson et al., 2008). In a recent report, the structure of the SPS from *Thermosynechococcus elongatus* was resolved, showing that this enzyme has 16 α-helices and 14 β-sheets, with UDP and S6P bound at the interface of the aforementioned A-and B-domains (Li et al., 2020). Whereas plant SPSs are specific for UDP-Gluc, cyanobacterial SPSs are not, and can accept other NDP-Gluc forms as substrates, such as ADP-Gluc and GDP-Gluc (Curatti et al., 1998; Lunn et al., 1999; Gibson et al., 2002). Another difference between cyanobacterial and plant SPSs is that the activity of the latter is regulated by light–dark modulation *via* reversible phosphorylation (Winter and Huber, 2000; Li et al., 2020).

**Figure 2:**
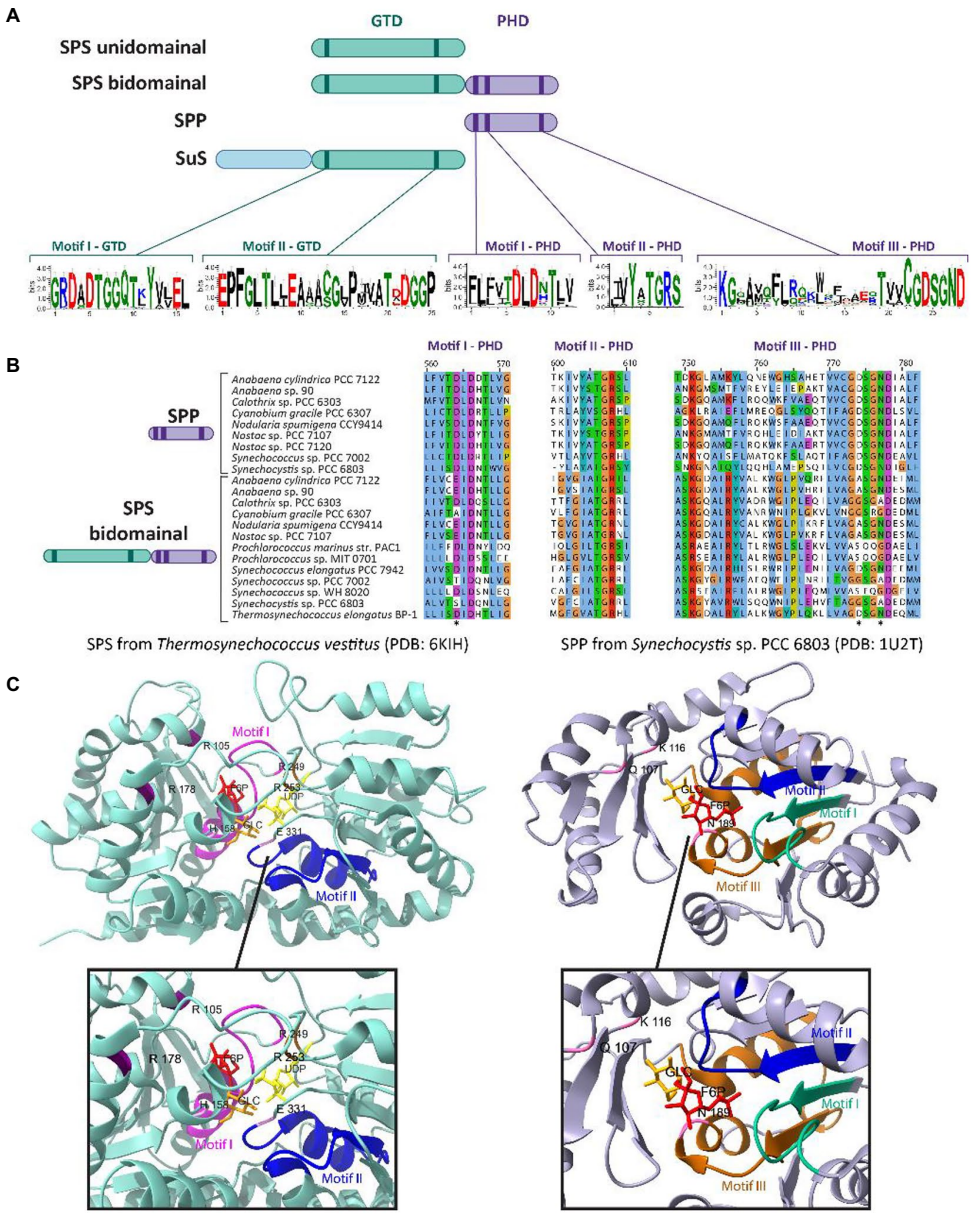
Conserved domains and motifs of SPS, SPP, and SuS among cyanobacterial species. **(A)** Schematic cartoon representing the domainal arrangements and the motifs present in SPS, SPP, and SuS. The glucosyl-transferase domain (GTD) is represented in green, while the phosphohydrolase domain (PHD) is represented in purple. The extended N-terminal domain found in SuS is represented in blue. GTD and PHD domains contain two and three conserved motifs, respectively. Logos for these conserved motifs were obtained using the WebLogo server (Crooks et al., 2004). **(B)** Multiple sequence alignment analysis of the deduced amino acid sequences for the three motifs present in the PHD domain. The alignment was performed using MEGA X (Kumar et al., 2018) and visualized with the Jalview multiple sequence alignment editor using the color scheme from ClustalX (Waterhouse et al., 2009). The asterisks indicate conserved residues that are mutated in the PHD domain in the sequences of cyanobacterial SPS bidomainal proteins relative to unidomainal homologs. **(C)** Crystal structure of the SPS from *T. vestivus* (PDB: 6KIH) and the SPP from *Synechocystis* sp. PCC 6803 (PDB: 1U2T) highlighting the motifs indicated in **(A)** and the residues involved in binding to their respective substrates (top) and a zoom-in of the catalytic centers of each enzyme (bottom). In the SPS (left panel), the residues R105, R178, R249 and R253 stabilize phosphate group of S6P; R249 and R253 stabilize the phosphate group of UDP; and H158 and E331 form hydrogen bonds with the 6-OH and 3-OH groups of glucose, respectively (Li et al., 2020). In the SPP (right panel), the residues Q107, K116, and N189 binds to S6P by hydrogen bonds in the glucose ring (Fieulaine et al., 2005). Figures were prepared with ChimeraX (Pettersen et al., 2021).

In cyanobacteria, the glucosyltransferase domain (GTD) of SPS contains two motifs that are highly conserved across glucosyl-transferase family enzymes (Figure 2A). Motif I (G-X_5_-GGQ-X_2_-Y-X_2_-EL) is located in the N-terminus of SPS and has been hypothesized to include residues necessary for defining the F6P binding site (Figure 2C, left panel; Ma et al., 2020). Motif II (E-X_7_-E) is highly conserved within the C-terminus of SPS and SuS enzymes; its flanking Glu residues play a catalytic role in the reaction by binding to UDP-Gluc (Figure 2C, left panel; Cumino et al., 2002; Ma et al., 2020; Kurniah et al., 2021). The first Glu residue of E-X_7_-E may function as the nucleophile, whereas the second Glu may function as the general acid/base catalyst (Cid et al., 2000). Both Glu residues in motif II are important for SPS activity, as demonstrated by point mutants in the GTD domain (E356A and E364A in SPS_7942_) of the bidomainal SPS encoded in *Synechococcus elongatus* PCC 7942 (SPS_7942_; see discussion below on bidomainal proteins) that disrupted sucrose synthesis, specifically preventing S6P formation (Liang et al., 2020).

#### 2.1.2. Sucrose phosphate phosphatase

The reversible reaction catalyzed by SPS is followed by the irreversible dephosphorylation of S6P to sucrose by SPP (Figure 1). SPP (EC 3.1.3.24) is a member of the L-2-haloacid dehalogenase (HAD) superfamily of aspartate-nucleophile hydrolases, belonging to the subfamily IIB that includes SPP from plants and cyanobacteria (Albi et al., 2016). SPP carries out the second step in sucrose synthesis by removing the phosphate group from S6P, forming sucrose (Lunn et al., 2000; Fieulaine et al., 2005). The hydrolytic activity of SPP is specific to S6P, showing little or no activity upon other sugar phosphates, such as F6P, which possesses a nearly identical phosphofructosyl moiety to S6P (Lunn et al., 2000). The mechanistic basis for the specificity of SPP to S6P against F6P appears to be related to the multiple active site contacts to the glucose ring, as revealed by a crystal structure of *Synechocystis* sp. PCC 6803 (Fieulaine et al., 2005).

Although members of the HAD superfamily generally have little overall sequence identity, they are characterized by three conserved motifs (I, II and III) related to the active site of the phosphohydrolase domain (PHD, Fieulaine et al., 2005; Figures 2A, B). All three motifs are highly conserved in SPP proteins among plants, algae, cyanobacteria, and mosses. Structurally, SPP proteins resemble a pair of “tongs” with a ‘core’ domain and a ‘cap’ domain connected by two flexible loop regions that act analogously to hinges between a closed (sucrose bound) and open (no ligand) enzyme state (PHD, Fieulaine et al., 2005). The conserved motifs that contribute to substrate binding line the interface between the two protein domains. Motif I, DXDX[T/V][L/V/I] (Figures 2A,B), is the most widely conserved among SPP sequences, and the first Asp is the functional nucleophile, which in the HAD phosphatase is transiently phosphorylated during the catalytic reaction (Collet et al., 1998; Fieulaine et al., 2005). The second Asp located in this motif is implicated in the acid–base catalysis reaction (Collet et al., 1998). Motif II, [S/T]X_2_, contains a Ser or Thr that is generally neighbored by hydrophobic residues, and functions to bind a phosphoryl oxygen in the substrate, orienting it in the correct position for nucleophilic attack by the first Asp in motif I (Wang et al., 2001). Motif III, KX_18-30_[G/S][D/S]X_3_[D/N] (Figures 2A,B), includes a conserved Lys that stabilizes the phosphorylated Asp intermediate state (Figure 2C, right panel). In addition, the two conserved Asp residues in this motif might form a system to direct water for the hydrolysis of the acyl-phosphate intermediate (Aravind et al., 1998). In a recent publication, the first Asp residue located in the motif I of the SPP domain of the bidomainal SPS_7942_ was mutated (D473A; Liang et al., 2020). This substitution inhibited sucrose synthesis, specifically the dephosphorylation of S6P to release sucrose, indicating that the Asp at position 473 is necessary for the SPP activity of the bifunctional SPS from this cyanobacterium.

In plants and several cyanobacterial species, the synthesis of sucrose is performed by a bidomainal SPS which encodes fused SPS and SPP domains on the same polypeptide (Curatti et al., 1998; Salerno and Curatti, 2003; Martinez-Noël et al., 2013; Kolman et al., 2015; Li et al., 2020; Supplementary Table S1). This is in contrast to many other cyanobacterial species where SPS and SPP are not fused, and are encoded by separate genes (Porchia and Salerno, 1996; Cumino et al., 2002; Lunn, 2002). In other words, two different domain arrangements have been described for cyanobacterial SPSs: (i) the minimal SPS unit with only a glucosyltransferase domain (GTD), found in filamentous cyanobacteria such as *Nostoc* sp. PCC 7119 (Porchia and Salerno, 1996), *Nostoc* sp. PCC 7120 (Cumino et al., 2002), and several species of unicellular cyanobacteria belonging to the genus *Gloeobacter*, *Thermosynechococcus*, and *Acaryochloris* (Blank, 2013); and (ii) the two-domain SPS prototype with both a GTD and a PHD, found in unicellular cyanobacteria such as *Synechocystis* sp. PCC 6803 (Curatti et al., 1998; Lunn et al., 1999), *Synechococcus* sp. PCC 7002 (Cumino et al., 2010) and *S. elongatus* PCC 7942 (Martinez-Noël et al., 2013; Figure 2A; Supplementary Table S1).

SPS was first reported in cyanobacteria based on characterization of a single functional GTD encoded in the filamentous cyanobacterium *Nostoc* sp. PCC 7119 (Porchia and Salerno, 1996). In this strain, two different isoforms of SPS can be found, SPS-I and SPS-II, both with similar molecular masses. The main difference between these two isoforms is their substrate specificity: whereas SPS-I has preference for UDP-Gluc, GDP-Gluc, and TDP-Gluc as substrates; SPS-II only uses UDP-Gluc and ADP-Gluc. It was previously accepted that unidomainal SPS enzymes were restricted to filamentous cyanobacterial species (Salerno and Curatti, 2003), but an extensive BLAST search in cyanobacterial genomes revealed that unidomainal SPSs are widespread in cyanobacteria, being present in species of *Gloeobacter*, *Thermosynechococcus*, *Acaryochloris*, a number of Nostocales, and other filamentous and unicellular cyanobacteria (Blank, 2013; Supplementary Table S1).

The first identification and characterization of cyanobacterial bidomainal SPS was reported in the unicellular cyanobacterium *Synechocystis* sp. PCC 6803 (Curatti et al., 1998). In independent research, SPS with fused GTD-PHD was also found in *Synechococcus* sp. PCC 7002 (Cumino et al., 2010). In addition to these species, bidomainal SPSs have been found in at least two filamentous species (*Nostoc punctiforme*, *Nodularia spumigena* CCY9414), and several unicellular cyanobacteria (e.g., *S. elongatus* PCC 7942, *Cyanobium* sp. PCC 7001, *T. elongatus* BP-1, several *Prochlorococcus* spp., and several *Synechococcus* spp.; Lunn, 2002; Martinez-Noël et al., 2013; Supplementary Table S1). Apart from a bidomainal SPS, *Synechocystis* sp. PCC 6803 has a separately encoded SPP enzyme (Lunn, 2002). The PHD of the bidomainal SPS lacks several of the conserved residues involved in the SPP function, including the critical Asp in motif I that is predicted to form an acyl-phosphate intermediate during the phosphatase reaction (Lunn, 2002; Fieulaine et al., 2005; Figure 2B). Other residues seem to be mutated in the PHD motif III of the bidomainal SPSs (indicated with an asterisk in Figure 2B), although their direct functions in SPP activity have not been described in the literature. The function of the PHD domain in biodomainal SPS is unknown, indeed some have shown these domains lack SPP activity, but it has been proposed that it might be involved in binding to newly synthesized S6P and transferring this molecule from the active site of SPS to the active site of the separately-encoded SPP in a form of metabolite channeling (Fieulaine et al., 2005). The presence of SPPs lacking enzymatic activity has also been reported in plants, where it has been suggested these non-functional protein play additional functions different from their canonical catalytic activity, for example as regulators (Albi et al., 2016).

#### 2.1.3. SPP-like proteins

**Figure 3:**
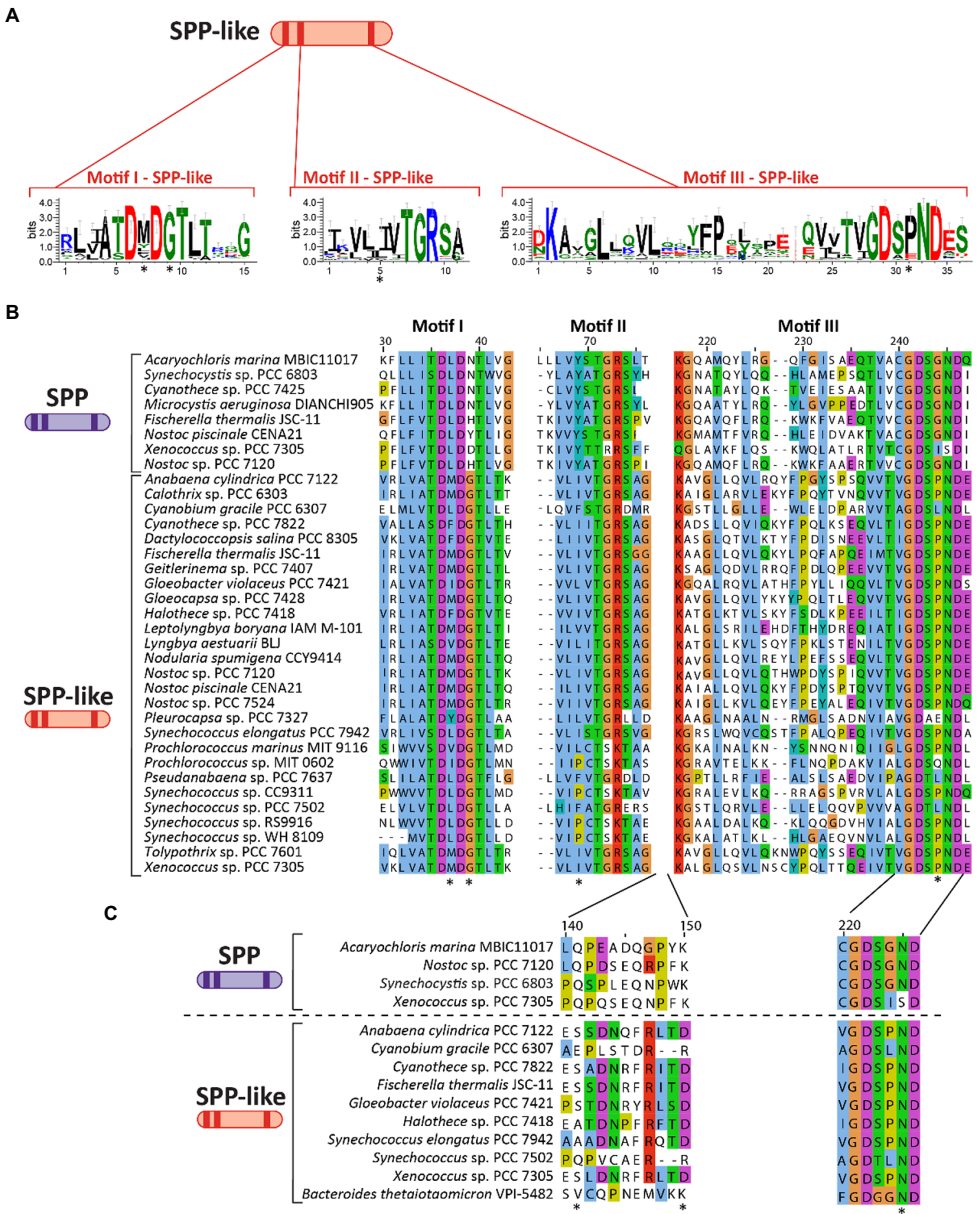
Primary structure and motifs of SPP-like proteins in cyanobacteria. **(A)** Schematic cartoon representing the motifs present in SPP-like proteins. Logos for these conserved motifs were generated using WebLogo server (Crooks et al., 2004). **(B)** Multiple sequence alignment analysis of amino acid sequences for three motifs found in SPP-like proteins, with asterisks denoting residues mutated in comparison with conserved sequence of cyanobacterial SPPs. **(C)** Multiple sequence alignment analysis of a region of amino acid residues between motif II and motif III previously implicated in S6P binding to SPP proteins. Asterisks indicate residues directly binding to the glucose ring of the S6P molecule by hydrogen bonds in a crystal structure reference (Li et al., 2020). The alignments in **(B,C)** were performed using MEGA X (Kumar et al., 2018) and visualized with the Jalview multiple sequence alignment editor using the color scheme from ClustalX (Waterhouse et al., 2009).

As previously discussed, SPP belongs to the class IIB subfamily of the HAD superfamily. Analysis of several cyanobacterial genomes revealed the existence of genes encoding homologous proteins of SPP (including some species encoding two or more distinct copies), but which have not been classified as SPP due to key distinctions in conserved domains. These SPP-like proteins are frequently annotated as (putative) HAD-superfamily hydrolases subfamily IIB, and while they contain the three motifs that define SPP (see above) they possess mutations in conserved residues in these motifs that distinguish them from classically defined SPPs (indicated with an asterisk in Figures 3A,B). For instance, SPP-like proteins present a conserved Gly in the fourth residue of motif I (Figure 3B) which is normally poorly conserved among cyanobacterial SPPs (Figure 2B). Conversely, the second residue of motif I is highly conserved as Leu in SPP (Figure 2B) but shows no clear conservation in SPP-like proteins (Figure 3B). Motif II seems to be conserved between SPP and SPP-like proteins, with the exception of the strictly-conserved Tyr residue in SPP which is not conserved in SPP-like proteins (Figures 2B, 3B). In motif III, all cyanobacterial SPPs have SGN as a X_3_ sequence at the end of this motif (Figure 2B), but SPP-like proteins do not maintain this sequence (Figure 3B). The first and third residues are conserved in most of the cases, but the second residue is not, with Gly substituted by Pro. Other important SPP residues include a Gln and a Lys located between motif II and III, and an Asn located in motif III, all of which are reported to participate in binding to the glucose ring of S6P *via* hydrogen bonds (Fieulaine et al., 2005; indicated by asterisks in Figure 3C).

**Figure 4:**
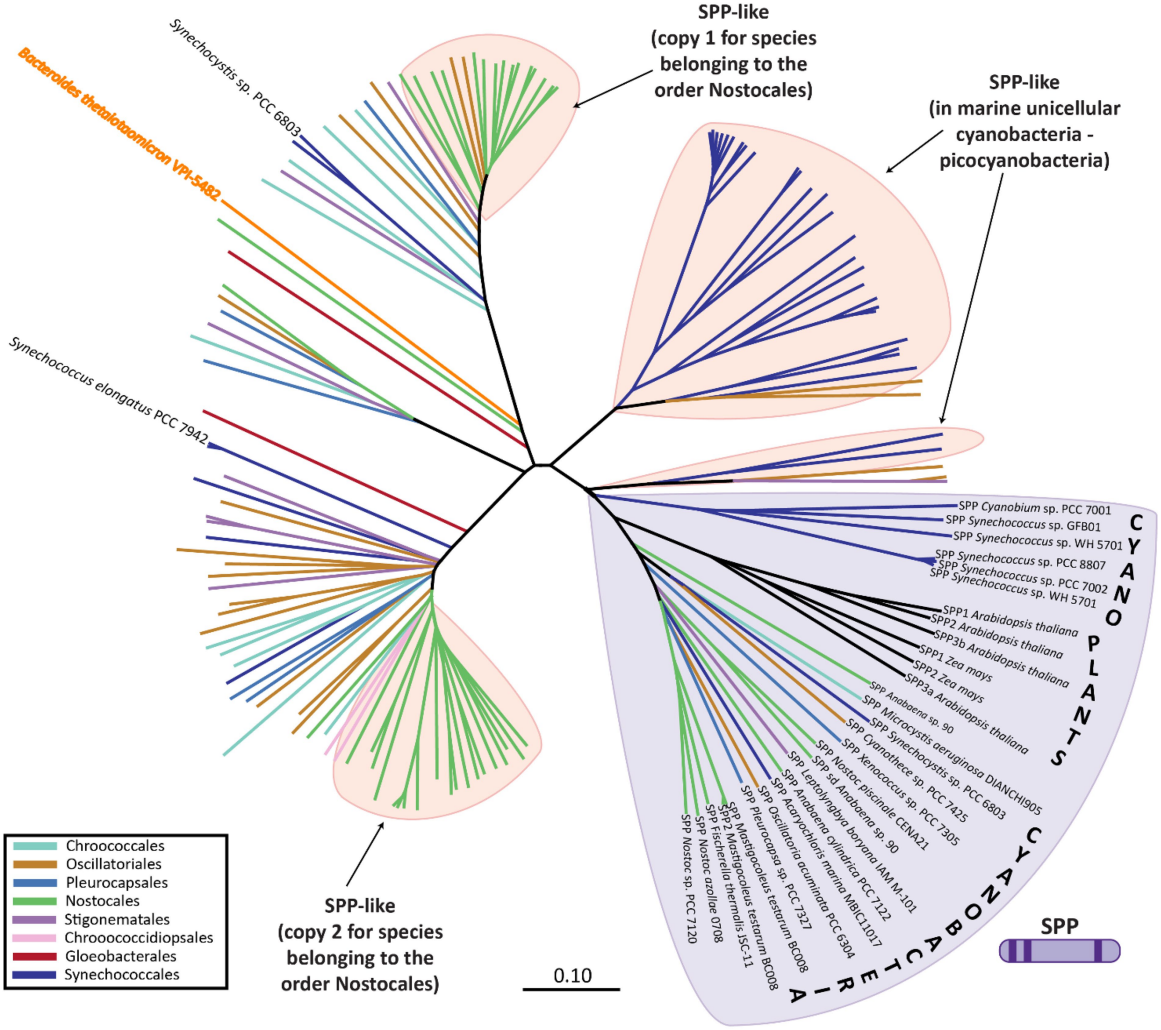
Phylogenetic analysis of SPP-like proteins encoded within different cyanobacterial species. SPP proteins are indicated by SPP followed by the name of the strain, whereas SPP-like are indicated only by the name of the strain. Unrooted neighbor-joining phylogenetic trees were constructed after sequence alignment of the SPP and SPP-like proteins using ClustalX with a BLOSSUM matrix and a bootstrap trial of 1,000. The graphical representation of the tree was generated using FigTree. Sequences were obtained from the non-redundant protein databases of the National Center for Biotechnology Information by BLAST searches. An extended version of this phylogenetic tree including all species names is available as Supplementary Figure S1.

Phylogenetic analysis of the SPP-like proteins encoded by cyanobacterial genomes revealed the existence of three main subclasses: (i) the first includes the SPP-like protein of *Synechocystis* sp. PCC 6803 and one of the two SPP-like protein paralogs encoded in the genome of members of the order Nostocales (among others); (ii) the second includes the SPP-like protein of *S. elongatus* PCC 7942 and the second of the two SPP-like protein paralogs encoded in the genome of members of the order Nostocales, and; (iii) SPP-like proteins that dominate in marine cyanobacteria, such as *Prochlorococcus* spp. (Figure 4; Supplementary Figure S1). In contrast to the conserved SPP-like subclasses, plant enzymes with verified SPP activity cluster closely to cyanobacterial classic SPPs (Figure 4; Supplementary Figure S1). For instance, the genome of *Arabidopsis thaliana* codifies four SPP isoforms, three of which exhibit SPP activity of varying catalysis rates, while the fourth one has no detectable activity (Albi et al., 2016). It has been suggested that the presence of SPP members with low/inactive catalytic activity might have regulatory functions instead (Albi et al., 2016), as it has been proposed for one of these SPP isoforms in sorghum seed germination (Jiang et al., 2015).

While canonical SPP proteins have been well reported in the literature, there exists much less direct evidence regarding the function of the SPP-like proteins. Only one member of the HAD subfamily IIB SPP-like proteins has been described in the literature, the enzyme BT4131 from the strictly anaerobic protobacteria, *Bacteroides thetaiotaomicron* VPI-5482 (Lu et al., 2005). As in the case of cyanobacterial SPP-like proteins, BT4131 is distantly related to SPP based on the phylogenetic analysis (Figure 4; Supplementary Figure S1). Substrate docking and biochemical experiments showed that BT4131 exhibited enzymatic activity on S6P and trehalose 6-phosphate, albeit with poor affinity and low rates of catalysis (Lu et al., 2005). Instead, BT4131 showed higher catalytic activity on cyclic hexose 6-phosphates and pentose 5-phosphates. To date, the function of these SPP-like proteins in cyanobacteria is unknown. One speculative possibility is that cyanobacterial SPP-like proteins play regulatory roles akin to those proposed for some plant homologs. However, it is equally possible that the aforementioned residue changes may influence or abolish the catalytic activity on S6P in these SPP-like proteins, or perhaps change their substrates entirely.

### 2.2. Sucrose degradation pathways

Sucrose is a compatible solute that is transiently synthesized and accumulated during periods of the salt-stress response across many cyanobacterial species. Catabolism of sucrose is therefore required to recycle the compatible solutes after relaxation of salt stress to avoid a net loss of carbon and energy (Baran et al., 2017). Three enzymes involved in sucrose breakdown have been identified in cyanobacteria: (i) invertase (Inv), which hydrolyzes sucrose directly into glucose and fructose; (ii) amylosucrase (AMS), which catalyzes the conversion of sucrose into fructose and glucose that is often transferred to maltooligosaccharides; and (iii) sucrose synthase (SuS), which uses (A/U)DP to reversibly split sucrose into (A/U)DP-Gluc and fructose (Liang et al., 2020; Figure 1).

#### 2.2.1. Invertases

The most broadly encoded pathway for sucrose degradation in cyanobacteria involves the enzyme Inv (EC 3.2.1.26), which irreversibly hydrolyzes sucrose into the monosaccharides, glucose and fructose (Figure 1; Supplementary Table S1). Phylogenetic analyses of Inv amino acid sequence data suggest that these enzymes originated from an ancestral Inv and their genes were transferred from cyanobacteria to plants, similarly to genes of other enzymes involved in sucrose metabolism (i.e., SPS and SPP, Vargas and Salerno, 2010). Invs are a large and diverse group of sucrose-cleaving enzymes, which can be classified partially based on their pH optimum: (i) acid Invs (Ac-Invs; β-fructofuranosidases) that possess a pH optimum in range from 4.5 to 5, and; (ii) alkaline/neutral Invs (A/N-Invs) which have a more-neutral pH optimum from 6.5 to 8 (Kirsch et al., 2019). A/N-Invs are not considered general β-fructofuranosidases since they are highly specific in catalyzing the cleavage of the α-1,2-glycosidic linkage of sucrose (Vargas et al., 2003; Vargas and Salerno, 2010; Xie et al., 2016; Liang et al., 2020). By contrast, Ac-Invs can cleave sucrose and other β-fructose-containing oligosaccharides such as raffinose and stachyose (Sturm, 1999). Bioinformatic analyses have shown that cyanobacterial genomes only encode A/N-Invs, but not Ac-Invs (Xie et al., 2016; Wan et al., 2018), whereas Ac-Invs can be mainly found in heterotrophic bacteria, yeasts, and plants (Tauzin and Giardina, 2014; Nadeem et al., 2015). In plants, A/N-Invs can be found in the cytosol, mitochondria, and/or in plastids, whereas Ac-Invs are frequently localized to the vacuolar space or bound to the cell wall (Tauzin and Giardina, 2014).

Early reports describing the hydrolysis of sucrose by A/N-Inv activity were published in *Trichormus variabilis* (Schilling and Ehrnsperger, 1985) and in *Scytonema* spp. (Page-Sharp et al., 1999). In addition, the first isolation and characterization of a cyanobacterial Inv was made in *Nostoc* sp. PCC 7120 (Vargas et al., 2003), which possesses two A/N-Inv encoded by *invA* and *invB* genes (Supplementary Table S1; Page-Sharp et al., 1999). By insertional inactivation, it has been demonstrated that InvA has a regulatory role controlling carbon flux from vegetative cells to heterocysts (López-Igual et al., 2010). The absence of this enzyme within the vegetative cells affects heterocyst differentiation due to a C/N imbalance in the filament, although it has been also speculated that sucrose or a product of its degradation might be regulating this process (Cumino et al., 2007; Vargas et al., 2011; Ehira et al., 2014). In addition, InvB activity is exclusively related to heterocysts, where it has an important function in heterocyst development, nitrogen fixation, and diazotrophic growth (López-Igual et al., 2010; Vargas et al., 2011; Xie et al., 2018). In a recent report, it was demonstrated that in *Synechocystis* sp. PCC 6803, the only enzyme responsible for *in vivo* sucrose degradation is an Inv (Kirsch et al., 2018).

#### 2.2.2. Amylosucrases

AMS (EC 2.4.1.4) is a glucosyltransferase that catalyzes the hydrolysis of the glycosidic bond in sucrose, leading to the release of glucose and fructose. Then, the released glucose is used to form α-1,4-linked linear insoluble glucans (amylose-like polymers; Potocki de Montalk et al., 2000; Figure 1). AMS belongs to glycoside hydrolase (GH) family 13 (the α-amylase family), and is organized in five domains: N, A, B, C (common to all proteins to the GH family 13), and an additional special domain called the B′ domain (only found in AMS; Skov et al., 2001). The A, B, and B′ domains form of an active site pocket, directly related to the activity of AMS (Skov et al., 2001). The first reported AMS in a cyanobacterium was in *Synechococcus* sp. PCC 7002, where sucrose synthesis genes (*sps* and *spp*) are grouped in the same transcriptional unit with fructokinase and AMS encoding genes (Perez-Cenci and Salerno, 2014). In comparison to Inv, it is relatively rare for cyanobacterial species to encode AMS, and the presence of this gene in the genome is frequently associated with the absence of other proteins able to breakdown sucrose (Supplementary Table S1).

#### 2.2.3. Sucrose synthases

SuS (EC 2.4.1.13) is a glucosyltransferase that can catalyze both the synthesis and cleavage of sucrose, but appears to be active principally in the cleavage reaction *in vivo* (Porchia et al., 1999; Curatti et al., 2002; Figure 1). The reversible cleavage of sucrose yields fructose and ADP-Gluc. SuS activity was first reported in cyanobacteria in *T. variabilis* ATCC 29413 (Schilling and Ehrnsperger, 1985) and *Nostoc* sp. PCC 7119 (Porchia et al., 1999). SuS has been mainly found in heterocyst-forming strains, where it seems to play an essential role in the control of carbon fluxes originating form vegetative cells through the cleavage of sucrose in the heterocysts (Porchia et al., 1999; Curatti et al., 2000, 2002, 2006, 2008). Outside of its roles in localizing the breakdown of sucrose in filamentous cyanobacteria, SuS is also reported in several unicellular cyanobacterial strains (Kolman et al., 2012; Figueroa et al., 2013; Tanabe et al., 2019; Supplementary Table S1). SuS has been implicated in the regulation of glycogen synthesis through its capacity to provide sugar nucleotide substrates (i.e., ADP-Gluc) required for elongation of α-1,4-glucoside chains (Cumino et al., 2002, 2007), a process that seems to be controlled by nutritional and environmental signals (Curatti et al., 2008).

Phylogenetic analysis suggests that a gene duplication of the GTD from a SPS-like gene and an addition of a N-terminal extension gave rise to SuS in most cyanobacteria (Cumino et al., 2002). These events took place before the branching of filamentous heterocyst-forming cyanobacteria. The occurrence of SuS in most-recently radiated cyanobacterial species, such as *Gloebacter violaceus* PCC 7421 and *Microcystis aeruginosa* NIES-4325, might be due to a more recent lateral gene transfer event (Blank, 2013; Tanabe et al., 2018, 2019).

## 3. Roles of sucrose in cyanobacteria

### 3.1. Sucrose as compatible solute

Cyanobacteria are ubiquitous organisms distributed widely across habitats and including terrestrial, aquatic, hypersaline waters, salt pans, and extreme environments such as deserts and hot thermal vents (Whitton and Potts, 2002). Cyanobacteria have evolved specific mechanisms to cope with the associated stress conditions of these ecosystems. In aquatic environments, salinity fluctuations are very common due to changes in freshwater inflow by climate, weather, and diurnal tidal currents. High salt concentrations promote loss in cytosolic water availability and increased ion concentrations that be destabilize many biomolecules (Klähn and Hagemann, 2011; Kolman et al., 2015; Kirsch et al., 2019). Cyanobacteria utilize the “salt-out strategy” for osmotic acclimation of the cytoplasm to changing salt concentrations (Pade and Hagemann, 2014). Briefly, the accumulation of small organic molecules called compatible solutes (including sucrose) acts to combat the loss of cytoplasmic water and corresponding drop in turgor pressure that normally accompanies a high extracellular osmotic pressure. In tandem, cyanobacteria engage numerous transporters that act to pump out the continuous influx of inorganic ions (e.g., Na^+^ and Cl^−^) that pass through the cell membrane under conditions of high external ionic pressure (Keshari et al., 2019).

Compatible solutes are organic molecules with low molecular masses without a net charge, which can accumulate to high (molar) concentrations in the cytoplasm without interfering with the cellular metabolism (Klähn and Hagemann, 2011). In cyanobacteria, different compatible solutes have been described and can be classified in the following substance classes: sugars (trehalose, sucrose), heterosides [glucosylglycerol (GG), glucosylglycerate (GGA)], amino acid derivatives (glycine betaine, glutamate betaine, homoserine betaine), polyols (glycerol), amino acids (proline), and organosulfurs (dimethylsulfoniopropionate; Kirsch et al., 2019; Kageyama and Waditee-Sirisattha, 2022). A correlation has been established between the class of the dominant compatible solute used by a given cyanobacterial species and its degree of exposure to salt stress within its natural habitat (Reed et al., 1984; Reed and Stewart, 1985). In general, freshwater strains with low halotolerance usually accumulate the disaccharides sucrose and/or trehalose as a compatible solute. Marine cyanobacteria accumulate the heterosides GG and GGA as osmolytes and are able to tolerate moderate salt concentrations. Finally, glycine betaine and glutamate betaine are preferentially synthesized as compatible solutes in halophilic species that inhabit extremely saline environments (Mackay et al., 1984; Hagemann, 2011). However, there are few exceptions to this classification. Notably, the widespread marine picoplanktonic *Prochlorococcus* strains appear to utilize sucrose as the preferred compatible solute (Klähn et al., 2010). In addition, some *Prochlorococcus* and *Synechococcus* strains also synthesize glycine betaine as well as GGA (Klähn et al., 2010).

### 3.2. Control of sucrose synthesis and degradation enzymes by ions

Commonly, the activity of enzymes responsible for synthesis and degradation of compatible solutes are regulated directly by allosteric binding of specific ions (Page-Sharp et al., 1999; Marin et al., 2002; Kirsch et al., 2019; Liang et al., 2020). In *S. elongatus* PCC 7942, SPS_7942_ activity is regulated by inorganic ions, Na^+^ and Cl^−^, which activate the SPS domain of this bifunctional protein, but have relatively little impact on the enzymatic activity of the SPP domain (Liang et al., 2020). The same ion-induced SPS activation has also been observed in the closely related strain, *S. elongatus* PCC 6301 (Hagemann and Marin, 1999), and other unicellular strains, such as *Synechocystis* sp. PCC 6803 (Hagemann and Marin, 1999; Desplats et al., 2005) and *M. aeruginosa* PCC 7806 (Kolman and Salerno, 2016). Inv activity in *S. elongatus* PCC 7942 is also regulated in an ion-dependent manner, showing decreased catalysis under elevated ion concentrations. The inhibition of Inv by ions combines with ion-induced SPS activation, promoting overall intracellular sucrose accumulation (Liang et al., 2020). The same regulation of invertase has been described for *Synechocystis* sp. PCC 6803 (Kirsch et al., 2018), suggesting that this is a fairly widespread mechanism that contributes to sucrose accumulation under salt stress.

Gene expression of sucrose synthesis enzymes is also controlled in a salt-induced manner (Cumino et al., 2010; Perez-Cenci and Salerno, 2014; Kolman and Salerno, 2016). For example, *sps* gene expression is usually transcriptionally activated upon salt addition, promoting sucrose synthesis proportional to the severity of osmotic stress (Kirsch et al., 2019). In *Synechococcus* sp. PCC 7002, a salt treatment increased the transcript levels of *sps* and *spp*, genes that organized together in an operon with AMS and fructokinase (Cumino et al., 2010; Perez-Cenci and Salerno, 2014). Similarly, *M. aeruginosa* PCC 7806 also contains a sucrose gene cluster including *spsA*, *susA*, and *sppA* that are all stimulated by salt (Kolman and Salerno, 2016). The transcript levels of *susA* were also increased in *M. aeruginosa* PCC 7806 and *G. violaceus* PCC 7421 cells after a salt treatment (Kolman et al., 2012). More recently, it was shown that transcription of the *sps* gene is upregulated after the addition of NaCl to *S. elongatus* PCC 7942 (Liang et al., 2020). In *Synechocystis* sp. PCC 6803, some sensors have been identified to be possibly related with perceiving and transducing signals of salt and hyperosmotic stresses (Marin et al., 2002; Shoumskaya et al., 2005; Liang et al., 2011), and a two-component response regulator was confirmed to control sucrose synthesis in *Nostoc* sp. PCC 7120 (Ehira et al., 2014). Finally, in some species, NaCl treatment has been shown to directly increase SPS specific activity and concurrently activate *sps* gene expression (Hagemann and Marin, 1999; Salerno et al., 2004).

### 3.3. Other functions of sucrose in cyanobacteria

Apart from its role as a compatible solute, sucrose acts in other cellular pathways. Sucrose and trehalose are considered major compatible solutes that enhance drought tolerance in cyanobacteria (Rajeev et al., 2013; Wang et al., 2018; Lin P. C. et al., 2020; Khani-Juyabad et al., 2022), though their functions in desiccation tolerance are less rigorously characterized. Sucrose also has well-established roles as a fixed carbon carrier molecule in some filamentous species, where it is produced in vegetative cells and catabolized in the heterocysts of nitrogen-fixing cyanobacterial species (Nürnberg et al., 2015). Sucrose acts a molecule to carry carbon and energy equivalents from vegetive cells to heterocysts, where it is consumed in part to drive the ATP-and NADH-requiring nitrogenase reactions (Juttner, 1983; Cumino et al., 2007; López-Igual et al., 2010; Vargas et al., 2011). It is proposed that sucrose transport primarily occurs through cell–cell septal junctions (Nürnberg et al., 2015).

Finally, it has been speculated that sucrose might also act as a signaling molecule in cyanobacteria (Desplats et al., 2005). In higher plants, sucrose metabolism is not only essential for the allocation of carbon resources but also participates in a regulatory network that coordinates metabolism and development (Curatti et al., 2006). Sucrose seems to be a versatile molecule with multiple roles in cyanobacteria, but most of them are poorly understood, raising the possibility that this sugar has underappreciated functions that remain unexplored.

## 4. Engineering cyanobacteria to produce sucrose

Innovations in biotechnology have taken advantage of aquatic photosynthetic organisms’ ability to create valuable products (e.g., lipids, antioxidants, pigments) to cope with environmental stressors (Chen et al., 2017; Morone et al., 2019). As a bioproduct naturally synthesized at high levels by some species of cyanobacteria in response to salt stress, sucrose has garnered attention for its potential as an alternative carbohydrate feedstock for higher-value goods (Hays and Ducat, 2015; Zhang et al., 2021). Sucrose generated by cyanobacteria could offer a number of advantages relative to plant-based feedstock crops, including potentially higher photosynthetic efficiencies and reduced requirements for potable water or arable land. Here, we review recent strategies employed to make cyanobacterial bioproduction of sucrose more productive and affordable.

### 4.1. Engineered heterologous transporters for sugar export

As discussed above, cyanobacteria can accumulate osmoprotectants up to hundreds-of-millimolar concentrations when exposed to hypersaline conditions (e.g., sucrose, trehalose, GG; Hagemann, 2011; Klähn and Hagemann, 2011). For instance, under moderate salt stress (200 mM NaCl), the common freshwater model cyanobacterium *S. elongatus* PCC 7942 accumulates nearly 300 mM intracellular sucrose (calculated based on a culture volume basis), representing a significant portion of the cell biomass (Suzuki et al., 2010). Although this degree of metabolite production presents an industrial and agricultural opportunity, cytosolic volume constrains how much sucrose can be accumulated: the costs associated with cyanobacterial cell recovery, lysis, and processing would likely exceed the economic value of the commodity products contained in the cytosol (Prabha et al., 2022). Therefore, secreting sugars into the supernatant for collection has been proposed as a more financially viable strategy. For this purpose, cyanobacteria have been engineered to express heterologous transporters capable of exporting lactate and hexoses (Niederholtmeyer et al., 2010; Angermayr et al., 2012).

Similarly, *S. elongatus* PCC 7942 was originally engineered to export sucrose by expressing sucrose permease (*cscB*) from *Escherichia coli* ATCC 700927 (Ducat et al., 2012), and multiple cyanobacterial species have since been similarly modified by different research teams (Table 1). In its native host, CscB is a sucrose/proton symporter that typically operates by utilizing the free energy of the proton gradient to import both molecules (Vadyvaloo et al., 2006). By contrast, during periods of cyanobacterial sucrose synthesis, internal sucrose concentrations greatly exceed external levels causing reversal of chemical gradients and driving sucrose export through CscB instead. CscB-expressing, sucrose-exporting *S. elongatus* PCC 7942 strains can secrete up to 80% of photosynthetically fixed carbon as sucrose, diverting these resources away from the accumulation of cellular biomass (Ducat et al., 2012). Although efforts to scale-up cyanobacterial sucrose production have not yet come to fruition (e.g., Proterro; Aikens and Turner, 2013), it has been estimated that such cyanobacterial strains have the potential to produce comparable amounts of sugar to traditional plant-based carbohydrate feedstocks. Realizing the promise of cyanobacterial sucrose is likely to require efforts to address problems of cyanobacterial/microalgal cultivation (beyond the scope of this review, but see Su et al., 2017; Khan et al., 2018) as well as strategies to maximize bioproduction rates.

**Table 1:** Productivity and genetic modifications of sucrose-producing cyanobacteria.

Species^a^	Overexpressed^b^^,^^*^	Downregulated^c^	Maximum productivity^d^	Salt for SPS	Sucrose promoter	Reference
*Syn*7002	−	−	24 mol 10^−17^ cells^e^	1 M NaCl	−	Xu et al. (2013)
	−	*glgA-I, glgA-II*	71 mol 10^−17^ cells^e^	1 M NaCl	−	
*Syn*6803	*cscB, sps_6803,_ spp_6803,_ ugp*	*ggpS*, *ggtCD*	0.69 mg L^−1^ h^−1^^f^	400 mM NaCl	P*_petE_*	Du et al. (2013)
	*sps_6803,_ spp_6803,_ ugp*	*ggpS*	3.1 mg L^−1^ h^−1^^f^	600 mM NaCl	P*_petE_*	
*Syn*7942	*cscB*	−	3.6 mg gDW^−1^ h^−1^	200 mM NaCl	−	Duan et al. (2016)
	*cscB, sps*_7942_	−	6.2 mg gDW^−1^ h^−1^	200 mM NaCl	P*_trc_*	
*Syn*7942	*−*	Synpcc7942_1125	5.9 mg L^−1^ OD_730_^−1^ h^-1e^	150 mM NaCl	−	Qiao et al. (2019)
	−	*manR* (Synpcc7942_1404)	6.7 mg L^−1^ OD_730_^−1^ h^-1e^	150 mM NaCl	−	
*Syn*7942	*cscB, sps*_6803_	−	5.6 mg L^−1^ h^−1^	−	P*_cpcB_*	Dan et al. (2022)
	*cscB, sps*_6803,_ *glgP*	−	6.9 mg L^−1^ h^−1^	−	P*_cpcB_*	
*Syn*6803	*cscB, sps_6803_*	*ggpS*	6.3 mg L^−1^ h^−1^	400 mM NaCl	P*_trc_*	Thiel et al. (2019)
*Syn*7942	*cscB, sps*_7942,_ *glgC*	−	8 mg L^−1^ h^−1^	150 mM NaCl	P*_trc_*	Qiao et al. (2018)
*Syn*6803	*cscB, sps_6803,_ spp_6803,_ ugp*	*invA* (sll0626), *ggpS, ggtCD*	10.1 mg L^−1^ h^−1^	200 mM NaCl	P*_petE_*	Kirsch et al. (2018)
*Syn*7942	*cscB*	−	10.4 mg L^−1^ h^−1^	150 mM NaCl	−	Löwe et al. (2017)
*Syn*7942	*cscB*	−	11 mg L^−1^ h^−1^	150 mM NaCl	−	Li C. et al. (2022)
*Syn*7942	*cscB*	−	16.7 mg L^−1^ h^−1^	106 mM NaCl	−	Hays et al. (2017)
*Syn*7942	*cscB, sps*_6803_	−	30 mg L^−1^ h^−1^	−	P*_trc_*	Abramson et al. (2016)
*Syn*2973	*cscB*	−	24.6 mg L^−1^ h^−1^	150 mM KCl	−	Song et al. (2016)
			35.5 mg L^−1^ h^−1^	150 mM NaCl	−	
*Syn*7942	*cscB*	−	28 mg L^−1^ h^−1^	150 mM NaCl	−	Ducat et al. (2012)
	*cscB*	*invA, glgC*	36.1 mg L^−1^ h^−1^	150 mM NaCl	−	
*Syn*7942	*cscB, sps*_6803,_ *rpaB*	−	48 mg L^−1^ h^−1^	−	P*_trc_*	Abramson et al. (2018)
*Syn*2973	*cscB, sps*_6803,_ *spp*_6803_	−	22.2 mg L^−1^ h^−1^	−	P*_trc1O_*, induced	Lin P. C. et al. (2020)
			47.2 mg L^−1^ h^−1^	−	P*_trc1O_*, uninduced	
	*cscB*	−	79.2 mg L^−1^ h^−1^	150 mM NaCl	−	

a *Syn7002, Synechococcus* sp. PCC 7002; *Syn7942*, *Synechococcus elongatus* PCC 7942; *Syn2973*, *Synechococcus elongatus* UTEX 2973; *Syn6803*, *Synechocystis* sp. PCC 6803.

b *cscB*, sucrose permease; *glgC*, ADP-glucose pyrophosphorylase; *glgP*, glycogen phosphorylase; *rpaB*, regulator of phycobilisome-associated B; *spp*, sucrose phosphate phosphatase; *sps*, sucrose phosphate synthase; *ugp*, UDP-glucose pyrophosphorylase.

c Genes are down-regulated or knocked out; *ggpS*, glucosylglycerol (GG)-phosphate synthase; *ggtCD*, GG transport system permease; *glgA-I/glgA-II,* glycogen synthase; *glgC,* ADP-glucose pyrophosphorylase; *invA*, invertase; *manR* (Synpcc7942_1404), manganese sensing response regulator; Synpcc7942_1125, histidine-containing phosphotransfer.

d Approximated extracellular sucrose values provided or calculated from titers.

e Intracellular sucrose yields.

f Intracellular and extracellular (total) sucrose yields.

* Subscript in *sps* and *spp* indicates the strain that it comes from (i.e., 6803 for *Synechocystis* sp. PCC 6803, 7942 for *S. elongatus* PCC 7942).

### 4.2. Increasing metabolic flux to sucrose pathways

Published strategies for improving rates of cyanobacterial sucrose productivity generally fall into two related strategies: increasing carbon flux towards the synthesis of sucrose through the upregulation of relevant biosynthetic activities, or by reducing the loss of carbon to competing pathways or sucrose reuptake. Perhaps the most straightforward approach for improving sucrose titers has been the overexpression of genes related to sucrose biosynthesis. Several studies have now found that flux leading to sucrose production can be most impacted by increasing the activity of SPS (Du et al., 2013; Duan et al., 2016; Lin P. C. et al., 2020), which is largely intuitive given that this enzyme catalyzes a commitment step to sucrose biosynthesis. Significant increases in sucrose production can be found in strains overexpressing SPS, even without allowing for sucrose export. First reported in *Synechocystis* sp. PCC 6803, a strain engineered to overexpress its native SPS (SPS_6803_) accumulated nearly twice as much intracellular sucrose than its wild-type counterpart (Du et al., 2013). Likewise, when the native SPS in *S. elongatus* PCC 7942 was overexpressed, internal sucrose concentrations were 93% higher than in wild-type (Duan et al., 2016). In addition, pairing SPS overexpression with sucrose export further increases total sucrose yields. When SPS_7942_ and CscB were co-overexpressed in *S. elongatus* PCC 7942, there was a 74% increase in sucrose compared to the CscB-only strain (Table 1; Duan et al., 2016), yet the nature of the SPS homolog that is overexpressed can strongly influence the degree to which sucrose production is improved. SPS_7942_ is bidomainal and bifunctional (i.e., possessing active GTD and PHD domains), in contrast to SPS_6803_ which is also bidomainal, but has a non-functional PHD domain and is regulated distinctly from SPS_7942_ (Curatti et al., 1998; Lunn et al., 1999; Gibson et al., 2002). However, the partial-functionality of SPS_6803_ does not mean it is less effective, as heterologous co-overexpression of SPS_6803_ and CscB in *S. elongatus* PCC 7942 increases sucrose production relative to overexpression of the native SPS_7942_ (Abramson et al., 2016; Dan et al., 2022; Table 1). It is curious that SPS_6803_ is a more effective enzyme for rerouting carbon flux towards sucrose bioproduction, given that it lacks a functional SPP domain (*S. elongatus* PCC 7942 encodes other endogenous SPP proteins in the examples above), so it is possible that this observation is related either to the manner in which salt-ions can regulate the function of some SPS domains (Liang et al., 2020), or to other unknown functions for SPP and/or SPP-like domains other than S6P phosphatase activity (see 2.1.3. SPP-like proteins).

While the overexpression of SPS has yielded substantial improvements, this strategy has not been equally successful with other proteins in the sucrose biosynthetic pathway. Overexpression of SPP from *Synechocystis* sp. PCC 6803 (SPP_6803_) either had no effect on, or decreased sucrose productivity in *S. elongatus* PCC 7942 or *Synechococcus elongatus* UTEX 2973 (Du et al., 2013; Lin P. C. et al., 2020). Similarly, overexpression of UDP-Gluc pyrophosphorylase (UGP), the protein producing UDP-Gluc as a substrate for SPS, led to less sucrose secretion (Ducat et al., 2012; Du et al., 2013). Only when these three enzymes were overexpressed simultaneously (i.e., SPS_6803_, SPP_6803_, and UGP), were sucrose levels increased in comparison with SPS_6803_-only strain, albeit marginally (Du et al., 2013; Table 1).

Another successful approach for improving the flux of carbon to sucrose biosynthesis is to accelerate the rate of the upstream carbon supply from the CBB. Multiple strains of cyanobacteria have been engineered to secrete sucrose through the heterologous expression of *cscB*, but the highest yields to-date have been obtained from strains with a more rapid metabolism and higher light tolerance relative to classic laboratory models (Table 1). *S. elongatus* UTEX 2973 is a recently re-characterized species that is 99.99% identical to *S. elongatus* PCC 7942, but has a doubling time as fast as ~2 h (compared to ~5–9 h for *S. elongatus* PCC 7942), and is more tolerant of high-light and high-temperature conditions (Kratz and Myers, 1955; Yu et al., 2015; Adomako et al., 2022). Expression of *cscB* in *S. elongatus* UTEX 2973 led to the development of strains with relatively high sucrose productivities (Song et al., 2016; Lin P. C. et al., 2020). A high sucrose titer was originally reported in such strains when exposed to 150 mM NaCl, reaching approximately 80 mg L^−1^ (Song et al., 2016). Lin et al. also created a *S. elongatus* UTEX 2973-*cscB* strain, and observed an even greater sucrose titer at 8 g L^−1^ at 150 mM NaCl, averaging out to 1.9 g L^−1^ day^−1^, over 2-fold higher than the productivities of *S. elongatus* PCC 7942, representing the highest sucrose titer published thus far (Lin P. C. et al., 2020), and illustrating the potential benefits of utilizing fast-growing strains that can reach higher densities.

Somewhat surprisingly, activation of the sucrose export pathway itself has been reported to increase the overall photosynthetic flux in some cyanobacterial strains. In *S. elongatus* PCC 7942, when sucrose synthesis pathways are placed under inducible promoters, a variety of enhancements in features related to photosynthesis have been reported in the hours following activation of the pathway (Ducat et al., 2012; Abramson et al., 2016; Santos-Merino et al., 2021b; Singh et al., 2022). The quantum efficiency of photosystem II, rate of oxygen evolution, relative rate of electron flux through the photosynthetic electron transport chain, oxidation status of photosystem, and rate of carbon fixation are all increased (Ducat et al., 2012; Abramson et al., 2016; Santos-Merino et al., 2021b). The latter observation is correlated with an increase in Rubisco abundance that was revealed by proteomic analysis >24 h following induction of sucrose export, and a concomitant increase in carboxysome number (Singh et al., 2022). While the mechanisms underlying these changes in photosynthetic performance are not well understood, it has been hypothesized that they arise from a relaxation in “sink limitations” on photosynthesis that can arise when the downstream consumption of products of photosynthesis (e.g., ATP, NADPH, CBB outputs) is insufficient to keep up with the supply (Santos-Merino et al., 2021a). Stated differently, when carbon fixation is not the rate-limiting step of cell metabolism (e.g., under enriched CO_2_ atmospheres commonly used in laboratory conditions), the expression of a heterologous pathway may act as an additional “sink” and bypass downstream limitations of cell growth and division. While this remains a speculative possibility, the relaxation of acceptor-side limitations on photosystem I suggests that sucrose secretion pathways (or other heterologous metabolic sinks) may utilize “excess” light energy that might otherwise be lost to photosynthetic inefficiencies under certain conditions (Abramson et al., 2016; Santos-Merino et al., 2021b). Uncovering the mechanisms underlying the photosynthetic phenotypes coupled to sucrose export might allow even greater improvements in photosynthesis and/or sucrose bioproduction.

### 4.3. Reducing metabolic flux to competing pathways

The alternative strategy to boost sucrose production is to improve the pool of sucrose or sucrose precursors by reducing flux to pathways that compete with sucrose biosynthesis for either substrates or total carbon pools. A straightforward example is to eliminate the dominant route for sucrose breakdown, such as the Inv proteins that are a dominant route of sucrose hydrolysis in many cyanobacterial models. In a recent report, inactivation of the *Synechocystis* sp. PCC 6803 invertase increased accumulated sucrose by 10-fold in both salt and salt-free conditions (Kirsch et al., 2018). These results were of higher magnitude, but similar trajectory to reports in other cyanobacteria, such as in sucrose-exporting *S. elongatus* PCC 7942 where a Δ*invA* background exhibited a 15% increase in extracellular sucrose (Ducat et al., 2012).

Glycogen is a storage molecule of cyanobacteria that is a significant alternative carbon sink, yet inhibiting glycogen synthesis has yielded variable results on sucrose secretion. For example, knockout of the two glycogen synthase genes (*glgA-I* and *glgA-II*) of *Synechococcus* sp. PCC 7002 led to an accumulation of three times more sucrose than wild-type under hypersaline conditions (Xu et al., 2013; Table 1). However, when another glycogen synthesis gene, ADP-glucose pyrophosphorylase (*glgC*), was downregulated in sucrose-secreting *S. elongatus* PCC 7942, there was only a minor or insignificant increase in sucrose (Qiao et al., 2018). GlgP is responsible for hydrolyzing glycosidic bonds in glycogen to release glucose-1-phosphate, so it was theorized that increasing GlgP activity would mobilize carbon from the glycogen pool for sucrose biosynthesis. However, when GlgP was overexpressed in sucrose-secreting strains of *S. elongatus* PCC 7942 with its native SPS, there were no changes in glycogen content and a decrease in sucrose was observed (Ducat et al., 2012; Dan et al., 2022), while heterologous expression of both SPS_6803_ and GlgP overexpression reduced glycogen content while increasing sucrose secretion by 2.4-fold (Dan et al., 2022). The variability in sucrose production of glycogen-deficient strains might be related to the pleotropic cellular deficiencies of these strains, including reduced growth, reduced O_2_ evolution and consumption, abnormal pigmentation, and light sensitivity (Suzuki et al., 2010; Ducat et al., 2012; Gründel et al., 2012; Xu et al., 2013; Qiao et al., 2018). These phenotypes align with a potential broader role for glycogen beyond carbon storage, which may include buffering against periods of starvation, oxygenic stress, high-light stress, salt stress, or diurnal/transient changes in light availability (Luan et al., 2019; Shinde et al., 2020). Given the increasing recognition of regulatory roles of glycogen, more nuanced strategies may be required to regulate the flux of carbon towards glycogen synthesis in order to reliably improve sucrose bioproduction (Huang et al., 2016).

In some cyanobacterial strains that utilize other compatible solutes as the dominant metabolite for osmoprotection, synthesis of these osmoprotectant compounds may compete with sucrose biosynthesis. One example is, GG, the primary solute utilized by moderately halotolerant cyanobacteria such as *Synechocystis* sp. PCC 6803 (Klähn and Hagemann, 2011). When GG-phosphate synthase (GgpS), the enzyme that generates a GG precursor, was knocked out in *Synechocystis* sp. PCC 6803, increased flux of carbon to sucrose production was reported (Du et al., 2013; Kirsch et al., 2019; Thiel et al., 2019; Table 1). A GgpS mutant incapable of generating GG under salt stress instead accumulated nearly 1.5-fold more sucrose than wild-type, although these engineered strains also exhibited growth inhibition at lower salt concentrations that would be well tolerated by wild-type lines (Du et al., 2013).

While most studies focus on restricting metabolic pathways that consume cellular carbon resources, downregulation of processes that compete for reducing equivalents may also be an alternative approach to engineering strains with high-sucrose productivity. Flavodiiron proteins are part of cyanobacterial photoprotective systems that are engaged during periods of redox stress (e.g., fluctuating light) and can direct electrons from an over-reduced photosynthetic electron transport chain to oxygen (Allahverdiyeva et al., 2015). The flavodiiron-catalyzed reaction is essentially a water–water cycle that dissipates potential energy from reducing equivalents generated from photosynthetic light reactions, but this reaction appears to be important for preventing photodamage under dynamic light conditions (Allahverdiyeva et al., 2013). Knockout of flavodiiron proteins Flv1 and Flv3 in *S. elongatus* PCC 7942 could boost sucrose production in a *cscB/sps*_6803_ expressing background (Santos-Merino et al., 2021b). Furthermore, activation of sucrose secretion pathways could partially compensate for the loss of Flv1/Flv3 under transient light changes, further suggesting that heterologous metabolic sinks may have some limited ability to utilize “overpotential” on the photosynthetic electron transport chain (Santos-Merino et al., 2021b).

### 4.4. Altering regulatory networks to increase sucrose synthesis

Sucrose biosynthesis is a natural component of many cyanobacterial adaptive responses, so a deeper understanding of the regulatory networks that control this process could allow researchers to manipulate sucrose production in the absence of abiotic stressors. In this context, a couple of studies have reported promising improvements in sucrose secretion rates by altering cyanobacterial two-component regulatory proteins, although the specific mechanisms remain uncertain. In a screen of all two-component regulatory factors in *S. elongatus* PCC 7942, Qiao and colleagues identified genes indirectly linked to sucrose productivity, glycogen accumulation, and photosynthetic activity (Qiao et al., 2019). The partial deletion of ManR, a protein that plays a regulatory role in Mn^2+^ uptake (Ogawa et al., 2002; Yamaguchi et al., 2002; Zorina et al., 2016), increased sucrose by 60%, a complete knockout of Synpcc7942_1125 increased sucrose by 41% (Qiao et al., 2019; Table 1). In a separate study, overexpression of the two-component protein regulator of phycobilisome assembly B (*rpaB*) reproduced a growth-arrest phenotype in *S. elongatus* PCC 7942 (Moronta-Barrios et al., 2013), and increased sucrose secretion in a *cscB*-expressing background (Abramson et al., 2018; Table 1). It was suggested that the growth arrest restricted carbon flux to many downstream pathways that might otherwise compete for sucrose biosynthesis, though a more specific alteration in regulatory processes controlling sucrose synthesis could not be excluded. While the number of studies is still limited, two-component signaling pathways have so far proven to be a promising strategy to improve sucrose yields, though our mechanistic understanding for these phenotypes is far from complete.

## 5. Applications of sucrose production in cyanobacterial co-culture

While the biotechnological focus for cyanobacteria has predominantly been upon direct synthesis of high-value products (Ducat et al., 2011; Knoot et al., 2018), there is growing interest in utilizing sugar-producing cyanobacteria for indirect bioproduction. This approach involves the use of carbohydrate-secreting cyanobacteria that support the growth of co-cultivated heterotrophic microbes. Co-cultures become “one-pot” reactions where cyanobacteria specialize in photosynthetic metabolism to supply carbon to a heterotroph, which in turn performs the metabolic labor of converting the carbon to higher-value goods or services (Hays and Ducat, 2015; Ortiz-Reyes and Anex, 2022). In this section, we will cover the modular nature of synthetic microbial consortia designed using sucrose-secreting cyanobacteria, their applications, and their future opportunities and challenges.

### 5.1. Potential advantages of modular microbial platforms

Microbial bioproduction is now a well-recognized approach that harnesses metabolic diversity for synthesis of valuable chemicals (e.g., polymers, fuels, pharmaceuticals) as an alternative to traditional environmentally unsustainable processes (Tsuge et al., 2016; Wendisch et al., 2016; Liu and Nielsen, 2019; Zhong, 2020; Wu et al., 2021). Multiple decades of sustained investments in microbial research, prospecting, and genetic engineering have yielded a wealth of bacterial strains optimized to generate specific bioproducts. In some cases, efficient bioproduction of a target compound can be achieved by expressing relevant metabolic pathways in different microbial species. But there are also many examples where heterologously expressed metabolic pathways perform poorly due to other physiological features of a microbe that make it a non-optimal chassis (Calero and Nikel, 2019). For this reason, it is often non-trivial to re-engineer cyanobacterial metabolism for direct synthesis of a desired compound, which may stubbornly resist efforts to improve product titer (Savakis and Hellingwerf, 2015; Nagarajan et al., 2016; Lin and Pakrasi, 2019).

A modular approach for multi-species product synthesis offers the capacity to leverage species with the most compatible physiology and desirable endogenous pathways for a given biochemical transformation, thus bypassing metabolic limitations of one biological chassis. At least in theory, each member of a synthetic microbial consortium can be conceptualized as a “module” selected specifically to perform functions well-suited with organism’s abilities. In this context, cyanobacteria-heterotroph co-cultures can be rationally designed to retain the advantages of cyanobacterial metabolism (i.e., use of light/CO_2_ inputs, efficient carbon fixation) and paired with other microbes that have demonstrated efficiency in transforming simple carbohydrates into a desired end product. Additionally, because the co-culture output can be changed by swapping the “heterotrophic module” (i.e., organism), some steps to optimize synthesis for one product (e.g., improving cyanobacterial sucrose production) may be transferable to achieve enhanced synthesis across many distinct cyanobacteria-heterotroph pairings. In practice, sucrose-secreting cyanobacteria have already been used as the basis for engineered microbial communities with numerous heterotrophic species and for a variety of end products (Table 2), although a number of improvements will be required to make these co-cultures feasible for scaled application.

**Table 2:** Sucrose-based autotroph-heterotroph co-cultures and their products.

Sucrose Strain				Heterotroph Strain				
Species^a^	Genotype^b^^,^^*^	Maximum productivity^c^	Induction	Species^d^	Genotype^e^	Product^f^	Maximum productivity^g^	Reference
*Syn*7942	*cscB*	400 mg L^−1^ d^−1^	106 mM NaCl	*B. subtilis*	−	α-amylase	not quantified	Hays et al. (2017) ^†^
				*E. coli W*	*phaCAB*, Δ*cscR*	PHB	0.04 mg L^−1^ d^−1^	
*Syn*7942	*cscB*	34.2 mg L^−1^ d^−1^	N/A; physical encapsulation	*A. vinelandii*	Δ*nifL*	PHB	8 mg L^−1^ d^−1^	Smith and Francis (2017) ^‡^
*Syn*7942	*cscB*	27.4 mg L^−1^ d^−1^	150 mM NaCl	*A. vinelandii*	Δ*nifL*	PHB	3.8% DW d^−1^	Smith and Francis (2016) ^†^
*Syn*7942	*cscB*	0.5 mg L^−1^ d^−1^	170 mM NaCl	*H. boliviensis*	−	PHB	28.3 mg L^−1^ d^−1^	Weiss et al. (2017) ^‡^
*Syn*7942	*cscB*	102 mg L^−1^ d^−1^	150 mM NaCl	*P. putida* EM178	*cscRABY*, Δ*nasT*	PHA	2.3 mg L^−1^ d^−1^	Hobmeier et al. (2020) ^†^
*Syn*7942	*cscB*	250 mg L^−1^ d^−1^	150 mM NaCl	*P. putida* EM178	*cscAB*	PHA	23.8 mg L^−1^ d^−1^	Löwe et al. (2017)
*Syn*7942	*cscB*	108 mg L^−1^ d^-1**^	150 mM NaCl	*P. putida* EM178	*cscRABY*, Δ*nasT*	PHA	42.1 mg L^−1^ d^−1^	Kratzl et al. (2023)
*Syn*7942	*cscB*	45 mg L^−1^ d^−1^	100 mM NaCl	*R. glutinis*	−	DW	24.8 mg L^−1^ DW^−1^ d^−1^	Li et al. (2017) ^†^
						TFA	1.2 mg L^−1^ d^−1^	
*Syn*2973	*cscB*	96 mg L^−1^ d^−1^	150 mM NaCl	*E. coli* BL21(DE3)	*cscA, cscB, cscK, mcr*	3-HP	9.8 mg L^−1^ d^−1^	Ma et al. (2022) ^†^
*Therm*PKUAC	*cscB*	18.1 mg L^−1^ d^−1^	150 mM NaCl	*E. coli* BL21(DE3)	*efe*	ethylene	0.74 mg L^−1^ d^−1^	Cui et al. (2022)
*Syn*7942	*cscB*	10 mg L^−1^ d^−1^			*ispS*	isoprene	0.03 mg L^−1^ d^−1^	
*Cup*H16	*sps*_6803_, *spp*_6803_, *scrY*	18.1 mg L^−1^ d^−1^	0.3% arabinose	*E. coli* W	*vioABCDE*, *cscABK* Δ*cscR*	violacein	4.5 mg L^−1^ d^−1^	Nangle et al. (2020)
					*crtEBIY, cscABK ΔcscR*	β-carotene	4.8 mg L^−1^ d^−1^	
*Syn*2973	*cscB*	0.7 mg L^−1^ d^-1**^	150 mM NaCl	*Y. lipolytica*	*carB, carRP*	β-carotene	325 mg L^−1^ d^−1^	Zhao et al. (2022) ^‡^
				*P. putida* KT2440	*sfp, bpsA*	indigoidine	1.9 g L^−1^ d^−1^	
*Syn*7942	*cscB*	263.5 mg L^−1^ d^−1^	150 mM NaCl	*V. natriegens*	*tyr*	melanin	1.56 mg L^−1^ d^−1^	Li C. et al. (2022) ^†^
					*tal*	*p*-coumaric acid	8.75 mg L^−1^ d^−1^	
					*budABC*	2,3-butanediol	60 mg L^−1^ d^−1^	
					*ldh*	lactate	100 mg L^−1^ d^−1^	
*Syn6803*	*cscB, sps*_6803_, Δ*ggpS*	164.3 mg L^−1^ d^−1^	400 mM NaCl	*E. coli* W	*ΔcscR,* Inv, Parvi	*ε*-caprolactone	102.7 mg L^−1^ h^−1^	Toth et al. (2022)
*Syn*7942	*cscB, sh3l*	108 mg L^−1^ d^−1^	50 mM NaCl	*P. putida* S12	*cscA*, *hmfH, sh3d*	FDCA	~100% in 4 days	Lin T. Y. et al. (2020) ^†^^,^ ^‡^
*Syn*7942	*cscB*	240 mg L^−1^ d^−1^	100 mM NaCl	*P. putida* EM173	*cscRABY, dnt*	2,4-DNT degradation	22.7 mg L^−1^ d^−1^	Fedeson et al. (2020) ^‡^
						PHA	5.1 mg L^−1^ d^−1^	
*Syn7942*	*cscB, sps*_7942_	200 mg L^−1^ d^−1^	N/A	*E. coli* ATCC 8739	*ΔpflB, ΔfrdABCD, ΔmgsA, ΔnarG, ΔnapA, ΔnarZ, cscB, gtfA*	electricity	380 μW^***^	Zhu et al. (2022)
				*S. oneidensis*	*ΔnapA, glk, cscAKB*			
				*G. sulfurreducens*	−			

a *Cup*H16, *Cupriavidus necator* H16; *Syn*7942, *Synechococcus elongatus* PCC 7942; *Syn*2973, *Synechococcus elongatus* UTEX 2973; *Syn*6803, *Synechocystis* sp. PCC 6803; *Therm*PKUAC, *Thermosynechococcus elongatus* PKUAC-SCTE542.

b *cscB,* sucrose permease; *scrY*, sucrose porin; *sh3l*, SH3 ligand; *sps,* sucrose phosphate synthase; *spp*, sucrose phosphate phosphatase.

c Approximated values from axenic cultivations in conditions most similar to co-culture conditions.

d *A. vinelandii*, *Azotobacter vinelandii* AV3; *B. subtilis*, *Bacillus subtilis* 168; *E. coli*, *Escherichia coli*; *G. sulfurreducens*, *Geobacter sulfurrenducens* PCA; *H. boliviensis*, *Halomonas boliviensis*; *P. putida*, *Pseudomonas putida*; *R. glutinis*, *Rhodotorula glutinis*; *S. onedensis*, *Shewanella onedensis* MR-1; *Y. lipolytica, Yarrowia lipolytica* CLIB138; *V. natriegens*, *Vibrio natriegens*.

e *bpsA*, non-ribosomal peptide synthetase; *budABC*, 2,3-butanediol gene cluster; *carB*, phytoene dehydrogenase; *carRP*, bifunctional lycopene cyclase/phytoene synthase; *crtEBIY*, β-carotene biosynthesis cassette; *cscA*, sucrose hydrolase; *cscB*, sucrose permease; *cscK*, fructokinase; *cscR*, sucrose operon repressor; *cscY*, sucrose porin; *dnt*, dinitrotoluence degradation gene cluster; *efe*, ethylene-forming protein; *frdABCD*, operon encoding fumarate reductase; *glk*, glucokinase; *gtfA,* sucrose phosphorylase; *hmfH*, HMF/furfural oxidoreductase; Inv, *cscA* invertase gene with an N-terminal *pelB* leader sequence; *ispS*, isoprene synthase; *ldh*, D-lactate dehydrogenase; *mcr*, malonyl-CoA reductase; *mgsA*, methylglyoxal synthase; *narG*, *napA*, and *narZ,* nitrate reductases; *nasT*, nitrate response regulator; *nifL*, negative regulator of nitrogen fixation; Parvi, synthetic Baeyer–Villiger monooxygenase; *pflB,* pyruvate formate-lyase B; *phaCAB*, polyhydroxybutyrate synthesis operon; *sfp*, phosphopantetheinyl transferase; *sh3d,* SH3 domain; *tal*, tyrosine ammonia lyase; *tyr*, tyrosinase; *vioABCDE*, violacein biosynthesis cassette. Unless otherwise denoted by “Δ,” genes are heterologously expressed.

f 3-HP, 3-hydroxypropionic acid; DNT, dinitrotoluene; DW, cyanobacterial biomass dry weight; FDCA, 2,5-furandicarboxylic acid; PHA, polyhydroxyalkanoate; PHB, polyhydroxybutyrate; TFA, cyanobacterial total fatty acids.

g Approximated values provided or calculated from titers.

† Enhanced photoautotroph growth in co-culture.

‡ Implemented spatial control of co-culture.

* Subscript in *sps* and *spp* indicates the strain that it comes from (i.e., 6803 for *Synechocystis* sp. PCC 6803, 7942 for *S. elongatus* PCC 7942).

** Values from axenic cultivation prior to the introduction of heterotroph.

*** Maximum power output reported for the four-species consortium.

### 5.2. Cyanobacterial co-culture as a flexible platform for value-added products

At the time of this writing, the most common metabolic output reported from cyanobacteria-heterotroph co-cultures are polyhydroxyalkanoates (PHAs), a class of biological polymers with comparative qualities to petroleum-based plastics. PHAs have the advantage of being both compatible in blends with commonly used petroleum-based polymers while also exhibiting superior biodegradation properties (Boey et al., 2021; Mezzina et al., 2021). Additionally, some heterotrophic microbes utilize PHAs as an intracellular storage polymer and under stress conditions can naturally hyperaccumulate PHAs in excess of 80% of their dry cell mass (Leong et al., 2014; Lee et al., 2021), making these compounds an ideal test case for the division of labor between metabolic specialists, as outlined above. Polyhydroxybutyrate (PHB) is a PHA polymer that has been produced in cyanobacterial co-culture with three different heterotrophic species: *Azotobacter vinelandii*, *Halomonas boliviensis*, and *E. coli* W (Hays et al., 2017; Smith and Francis, 2017; Weiss et al., 2017; Table 2). PHB is a natural storage polymer for both *A. vinelandii* and *H. boliviensis*, while heterologous expression of the *phaCAB* operon in *E. coli* will confer PHB synthesis capability. The most productive co-cultures reported included a heterotrophic partner species that was naturally capable of PHB synthesis. Notably, the co-cultivation of *S. elongatus* PCC 7942 *cscB* with *H. boliviensis* was extended over 6 months with no organic carbon input, demonstrating that these synthetic consortia can be stable and productive over long time periods (Weiss et al., 2017).

*Pseudomonas putida* is a model organism that naturally accumulates medium chain length PHAs (mcl-PHAs) granules in response to starvation, primarily under low-nitrogen and high-carbon conditions (Hoffmann and Rehm, 2004). While sucrose is not naturally consumed by *P. putida*, expression of heterologous sucrose transporters and sucrose hydrolyzing enzymes allows it to grow on sucrose as the sole carbon source (Sabri et al., 2013; Löwe et al., 2020), a strategy that has been used to enable other microbial species without native pathways to consume cyanobacterially secreted sucrose (Sabri et al., 2013; Hobmeier et al., 2020; Zhang et al., 2020). Indeed, initial reports demonstrated that *P. putida* expressing *cscAB* was capable of growing solely on sucrose provided by *S. elongatus* PCC 7942 and accumulated PHA in co-culture, though sucrose utilization was incomplete and productivities were modest (Löwe et al., 2017; Fedeson et al., 2020). Additional expression of a sucrose porin (*cscY*) and a sucrose operon repressor (*cscR*) further improved sucrose utilization (Löwe et al., 2020), while further optimization of the nitrogen-deficiency response pathway (Hobmeier et al., 2020) and culture conditions could boost PHA titer further (Kratzl et al., 2023; Table 2).

Other co-culture products include the metabolites ethylene, isoprene, 3-hydroxypropionic acid (3-HP), and 2,3-butanediol (Table 2), which are compounds in a broader class of industrially relevant precursors widely used for chemical synthesis (e.g., diols, organic acids, gaseous alkenes; Cui et al., 2022; Li C. et al., 2022; Ma et al., 2022). In most of these reports, the heterotrophic microbe utilized were *E. coli* substrains, although the rapidly growing halophile *Vibrio natriegens* was able to produce a relatively high amount of 2,3-butanediol in co-culture (Li C. et al., 2022). Interestingly, co-cultures of *S. elongatus* PCC 7942 and *P. putida* designed to convert 5-hydroxymethylfurfural to 2,5-furandicarboxylic acid (FDCA), a common precursor molecule, exhibited higher efficiency when the two species were engineered to display complementary surface proteins (Lin T. Y. et al., 2020). The authors suggest that physical binding between the two species could improve metabolic exchange (Lin T. Y. et al., 2020), an intriguing strategy that may be valuable to develop further.

Beyond commodity products, several higher-value chemicals expand the metabolic repertoire of cyanobacteria-heterotroph co-cultures. The pigment industry makes routine use of a number of compounds that generate significant environmental hazards when chemical synthesis methods are used (Pereira and Alves, 2012). Biosynthetic pathways for pigment derivatives (e.g., indigoidine for the popular pigment, indigo) are being explored for more environmentally conscious pigment synthesis (Celedón and Díaz, 2021). Recently, co-cultures have been reported for the synthesis of indigoidine using the heterotroph *P. putida*, β-carotene with *E. coli* or the yeast *Yarrowia lipolytica*, and violacein by *E. coli* (Nangle et al., 2020; Zhao et al., 2022). Although most cyanobacteria-heterotroph co-cultures make use of the model laboratory strain *S. elongatus* PCC 7942, Zhao and colleagues used a sucrose-secreting variant of the fast-growing and high-light tolerant relative, *S. elongatus* UTEX 2973, in their co-culture experiments to produce indigoidine and β-carotene (Zhao et al., 2022). The cosmetic *p*-coumaric acid, is another higher-value compound useful for its antioxidant and antimicrobial properties (Boz, 2015; Boo, 2019). The biosynthetic pathway for *p*-coumaric acid was introduced into *V. natriegens* and co-cultures of these engineered strains with *S. elongatus* PCC 7942 allowed for photosynthetically driven *p*-coumaric acid production (Li C. et al., 2022). Other recent reports provide further evidence of the flexibility of this cyanobacterial co-cultivation system (see Table 2), including bioproduction of fatty acids (Li C. et al., 2022), *ε*-caprolactone (Toth et al., 2022), lactate (Li C. et al., 2022), and secreted enzymes (Hays et al., 2017). Additionally, some products can be used to feed downstream bacteria and develop more complex systems. A four-species consortium utilized lactate-consuming *Shewanella onedensis* to generate electricity and acetate, in which the latter was consumed by *Geobacter sulfurreducens* to produce CO_2_ for *S. elongatus* PCC 7942 (Table 2; Zhu et al., 2022).

One final co-culture example was constructed more in the service of remediating an environmental toxin, rather than producing a specific byproduct (Fedeson et al., 2020). *S. elongatus* PCC 7942 was co-cultured with an engineered strain of *P. putida* expressing a pathway for 2,4-dinitrotoluene (2,4-DNT) degradation (Akkaya et al., 2018; Table 2). 2,4-DNT is an environmentally stable and toxic byproduct generated from the manufacture of polyurethane, pesticides, and explosives (Griest et al., 1995; Ju and Parales, 2010). In order to prepare co-cultures that were stable in the face of toxic levels of 2,4-DNT, it was necessary to encapsulate sucrose-secreting *S. elongatus* PCC 7942 within an alginate hydrogel, which increased the resilience of the cyanobacteria to the environmental stress without diminishing its capacity to perform photosynthesis and secrete sucrose for *P. putida* consumption (Fedeson et al., 2020). Notably, the strategy of immobilizing one or more microbial partner in a hydrogel was utilized in a number of the aforementioned co-culture experiments (Smith and Francis, 2017; Weiss et al., 2017; Li X. et al., 2022; Zhao et al., 2022), and encapsulated cyanobacterial strains exhibited increased resilience to environmental stressors relative to planktonic controls, while simultaneously maintaining or increasing per-cell sucrose secretion rates.

### 5.3. Co-culture as a platform to study microbial communities

Phototrophs and heterotrophs are often metabolically intertwined in natural contexts (Morris, 2015; Henry et al., 2016). For example, many marine *Prochlorococcus* species secrete organic carbon to neighboring heterotrophic partners that perform functions in detoxifying reactive oxygen species present in the open ocean (Morris et al., 2011; Braakman et al., 2017). It has been hypothesized that the natural export of sugars from *Prochlorococcus* and other cyanobacteria may prime them to engage with surrounding heterotrophs *via* cross-feeding, and potentially “outsource” the metabolic burden of synthesizing some nutritional requirements to other organisms (Werner et al., 2014; Henry et al., 2016; Braakman et al., 2017). Natural microbial communities and symbiotic relationships usually develop over evolutionary time scales and may exhibit numerous and complex cross-feeding patterns and other self-stabilizing interactions (Konopka et al., 2015). Yet, these important dynamics can be challenging to study due to the difficulty of disentangling specific mechanisms from the complex interaction networks (Ponomarova and Patil, 2015). The fact that many natural symbioses also have cyanobacterial partners that exchange fixed carbon for other microbial partner (s) has led some groups to explore synthetic cyanobacteria/heterotroph co-cultures as a possible “bottom-up” system to gain insight into complex microbial consortia.

Synthetic phototroph-heterotroph microbial consortia may represent a complementary system to study natural consortia in parallel, as they present a platform for interrogating microbial interactions that is relatively simple, genetically tractable, and experimentally tunable (Table 3; De Roy et al., 2014; Song et al., 2015). One intriguing phenomenon that recurs across several synthetic cyanobacteria/heterotroph co-cultures is an increase in the vigor or productivity of one or both partners relative to axenic controls. For instance, cyanobacterial growth was enhanced in mixed culture with several heterotrophic species (Hays et al., 2017; Li et al., 2017; Hobmeier et al., 2020; Ma et al., 2022), although the partner species were evolutionarily “naïve” to one another. Similarly, heterotrophic productivity in co-culture can be significantly higher than can be attributed to the cyanobacterially secreted sucrose (Hays et al., 2017; Cui et al., 2022; Ma et al., 2022). Conversely, when cyanobacteria are allowed to overpopulate a synthetic co-culture, heterotrophic partners may exhibit reduced viability (Hays et al., 2017). It is highly likely that some of these effects arise due to unprogrammed metabolic interactions and emergent behaviors of division of labor (Rafieenia et al., 2022), such as the generation of damaging reactive oxygen species (Hays et al., 2017; Ma et al., 2022).

**Table 3:** Synthetic cyanobacteria-heterotroph microbial consortia used as a platform to study microbial interactions.

Sucrose strain^a^	Genotype^b^	Heterotroph strain^c^	Genotype^d^	Notes	Reference
*Syn*7942	*cscB*	*E. coli* K-12	AA knockouts	Utilizes metabolic modeling and experimental validation to predict co-cultivation outcomes and identify optimizable parameters.	Zuñiga et al. (2020)
		*E. coli* W	−		
		*Y. lipolytica*	SUC2		
		*B. subtilis*	−		
*Syn*7942	*cscB*, *sps*_6803_	*A. vinelandii*	Δ*nifL*	Develops tripartite consortium with carbon-providing *S. elongatus* PCC 7942 and nitrogen-providing *A. vinelandii* to support a third microbe. Performed computational analyses to identify bottlenecks to improve cultivation conditions.	Carruthers (2020)
		*E. coli* K-12 MG1655	*cscABK*		
		*C. glutamicum*	−		
		*B. subtilis* 168	−		
*Syn*2973	*cscB*	*E. coli* BL21(DE3)	*cscABK*, *mcr*	Utilizes transcriptomic, proteomic, and metabolomic analyses to reveal differentially regulated pathways during co-cultivation to identify optimizable parameters to improve stability and 3-hydroxypropionic productivity.	Ma et al. (2022)
*Syn*7942	*cscB*	*E. coli* MG1655	*cscABK*	Spatially separates subpopulations with encapsulation to impart species stability while still allowing the transport of small molecules.	Wang et al. (2022)
*Syn*7942	*cscB*, *sps*_6803_	*E. coli* W	Δ*cscR*	Utilizes individual-based modeling in spatial context to predict colony fitness.	Sakkos et al. (2022)
*Syn*7942	*cscB*, *sps*_6803_	*E. coli* W	Δ*cscR*	Integrates quorum sensing modules for cross-species communication.	Kokarakis et al. (2022)

a *Syn*7942, *Synechococcus elongatus* PCC 7942; *Syn*2973, *Synechococcus elongatus* UTEX 2973.

b AA knockouts, multiple one-way amino acid auxotrophs were generated; *cscB*, sucrose permease; *sps_6803_*, sucrose phosphate synthase from *Synechocystis* sp. PCC 6803.

c *A. vinelandii*, *Azotobacter vinelandii* AZBB163; *B. subtilis*, *Bacillus subtilis* 168; *C. glutamicum*, *Corynebacterium glutamicum* 13032; *E. coli*, *Escherichia coli*, *Y. lipolytica*, *Yarrowia lipolytica* Po1g.

d *cscABK*, sucrose utilization operon; *cscR*, sucrose operon repressor; *mcr*, malonyl-CoA reductase; *nifL*, negative regulator of nitrogen fixation; SUC2, cassette for internal and external invertases.

Our current understanding of the emergent properties of mixed microbial communities is limited and cannot fully explain observed phenomena. Preliminary analyses and multi-omics approaches have been used to predict hidden interactions within synthetic consortia, providing insight on areas of cooperation and competition that could be validated and exploited to design more robust co-cultures (Carruthers, 2020; Zuñiga et al., 2020; Ma et al., 2022). Synthetic co-cultures also present a simpler set of variables in comparison to natural communities which may be more amenable to simulations, such as agent-based modeling, for predicting emergent behaviors in a population (Sakkos et al., 2022). Finally, additional layers of metabolic exchange can be designed into the synthetic co-culture system to experimentally probe and validate hypotheses of inter-species exchange. A notable example in this regard is multiple groups’ use of the diazotroph, *A. vinelandii,* to fix atmospheric nitrogen and secrete ammonia, effectively creating a carbon-for-nitrogen exchange in co-culture with sucrose-secreting cyanobacteria (Smith and Francis, 2017; Carruthers, 2020). Taken together, the computational, systems, and genetic toolkits available for synthetic microbial consortia may lead to important insights on the dynamics of microbial exchange that would be difficult to probe in natural microbiomes.

## 6. Challenges and future perspectives

Cyanobacterial sucrose production exhibits considerable potential to facilitate sustainable bioproduction using light and CO_2_ but could benefit from still further enhancements in productivity. Expanding into more elaborate metabolic engineering efforts guided by cyanobacterial genome-scale metabolic models might be one approach to identify other potential metabolic targets to increase sucrose yields. In addition, only one transporter has been used so far to facilitate sucrose secretion in cyanobacteria, CscB. While this transporter seems to work properly in many cyanobacterial strains (Table 1), *Synechocystis* sp. PCC 6803 is an exception (Du et al., 2013; Kirsch et al., 2018). CscB has a relatively low affinity for sucrose (Sahin-Tóth and Kaback, 2000), so alternative transporters with higher affinity or transport kinetics might be used to boost cyanobacterial sucrose export, expand the range of cyanobacterial species that can be engineered, or used to increase the uptake rates for co-cultured heterotrophs.

Although enzymes involved in cyanobacterial sucrose synthesis and degradation have been the subject of extensive study, there are still major gaps in our understanding of the function of these enzymes. Areas that contain a number of open questions for future study include: (i) the co-evolution of bidomainal SPS with and without SPP activity among cyanobacterial species; (ii) the role (s) and substrate (s) of SPP-like proteins in cyanobacteria; and (iii) the alternative roles of sucrose in cyanobacteria apart from its osmoprotective functions. Increasing the knowledge in all these areas will not only be useful to understand the regulation and evolution of different sucrose enzymes in cyanobacteria, but also to further engineer these enzymes to obtain high sucrose yields.

Cyanobacteria hold considerable potential as cell factories to produce sucrose, yet the development of commercially viable applications of this strategy will require a significant amount of additional research and optimization. Importantly, while yields of sucrose from cyanobacteria could theoretically exceed production from traditional plant crops at scale, significant barriers to translate results from the lab to the field are evident. For instance, deployment of outdoor cultivation would require strains that exhibit resilience to the dynamic fluctuations of temperature, light, diurnal cycles, and abiotic stresses (Jaiswal et al., 2022). Furthermore, while the bioavailability of sucrose lends itself to a high degree of flexibility in the design of co-cultures, it also makes cyanobacterial cultures highly vulnerable to invasive microbes (Hays and Ducat, 2015; Gao et al., 2022). Contamination that reduces culture output would be highly likely in any scaled system without the implementation of aggressive confinement and/or pesticidal treatments that would greatly increase the cost of production. Alternatively, efficient, automated, economical, and sustainable systems to separate secreted sucrose might be employed, as explored in a recent membrane-filtration system (Hao et al., 2022).

A much tighter integration of the signaling and metabolic exchanges between cyanobacterial and heterotroph co-culture partners might suppress contaminating species through competition and exclusion. Adaptative laboratory evolution could be a useful strategy to domesticate increasingly stable co-cultures by better integrating and adapting the partners to one another (Konstantinidis et al., 2021). Rational engineering strategies to generate more intricate coordination of activities between species and at the population level might also contribute to this goal (Kokarakis et al., 2022). Cyanobacterial and heterotrophic partner species that have been more extensively designed to cooperate and coordinate would also be likely to exhibit higher end-product titers relative to the current productivities achievable from co-culture. The exploration of mechanisms that promote partner coordination in synthetic communities through rational and directed research efforts could provide additional insights into the underlying organizational principles in robust cyanobacterial symbioses that occupy many natural ecological niches.

## 7. Methods

### 7.1. Sequence homology

The protein sequences of the orthologues of the different enzymes involved in the sucrose biosynthesis and degradation were obtained from NCBI and Uniprot databases. These sequences were retrieved using BLAST tools in both databases; enzymes with a well-established role in these pathways in cyanobacteria were used a queries: SPS unidomainal from *Nostoc* sp. PPC 7120 (GenBank accession No. BAB76075.1; Cumino et al., 2002), SPS bidomainal from *S. elongatus* PCC 7942 (GenBank accession No. ABB56840.1; Liang et al., 2020), SPP from *Synechocystis* sp. PCC 6803 (GenBank accession No. BAA18419.1; Fieulaine et al., 2005), SuS from *Nostoc* sp. PCC 7120 (GenBank accession No. BAB76684.1; Ehira et al., 2014), AMS from *Synechococcus* sp. PCC 7002 (GenBank accession No. ACA98889.1; Perez-Cenci and Salerno, 2014) and from *Alteromonas macleodii* KCTC 2957 (Kolman and Salerno, 2016), and INV from *S. elongatus* PCC 7942 (GenBank accession No. ABB56429.1; Liang et al., 2020). For SPP-like proteins, we seeded the analysis using the sequence from *S. elongatus* PCC 7942 (GenBank accession No. ABB56598.1). Each protein in this query list was used to search for homolog sequences in the genome of 121 cyanobacterial genomes, and hits with an E-value less than or equal to 10^−15^, an identity less than or equal to 35% and a coverage less than or equal to 80% were considered true homologs.

### 7.2. Multiple sequence alignments

Multiple sequence alignment analyses were performed using MEGA X (Kumar et al., 2018) and visualized with the Jalview multiple sequence alignment editor using the color scheme from ClustalX (Waterhouse et al., 2009). Logos for the conserved motifs for each analyzed enzyme were obtained using WebLogo server (Crooks et al., 2004).

### 7.3. Phylogenetic trees

Unrooted neighbor-joining phylogenetic trees were generated using MEGA X after the multiple sequence alignments of the sequence of SPP proteins and SPP-like proteins using ClustalX with a BLOSSUM matrix and a bootstrap trial of 1,000. The graphical representations of the trees were created using FigTree. The neighbor-joining tree of SPP-like and SPP sequences was generated using the *p*-distance substitution method including both transitions and transversions, uniform rates among sites, and pairwise deletion treatment. Support for each node was tested with 1,000 bootstrap replicates.

### 7.4. Protein structure analysis

The previously published crystal structures of SPS from *Thermosynechococcus vestivus* (Li et al., 2020) and SPP from *Synechocystis* sp. PCC 6803 (Fieulaine et al., 2005) were downloaded from PDB (Berman et al., 2003) with IDs 6KIH and 1U2T, respectively. All structure figures were prepared using ChimeraX (Pettersen et al., 2021).

## Author contributions

MS-M, LY, and DD outlined the scope and content of the manuscript. MS-M and LY conducted the literature review and wrote the draft manuscript. MS-M conducted the phylogenetic analyses and prepared Figures 1–4 and Supplementary Table S1. LY prepared the Tables within the main manuscript, while MS-M prepared the tables in the Supplemental material. MS-M, LY, and DD reviewed, edited, and proofed the manuscript. All authors contributed to the article and approved the submitted version.

## Funding

This work was primarily supported by the Department of Energy and Basic Energy Sciences Division (Grant: DE-FG02-91ER20021), and the National Science Foundation and the Division of Molecular and Cellular Bioscience (Grant: 1845463). LY was supported by a fellowship from the Plant Biotechnology for Health and Sustainability Training Program at Michigan State University (Grant: NIH T32-GM110523).

## Conflict of interest

The authors declare that the research was conducted in the absence of any commercial or financial relationships that could be construed as a potential conflict of interest.

## Publisher’s note

All claims expressed in this article are solely those of the authors and do not necessarily represent those of their affiliated organizations, or those of the publisher, the editors and the reviewers. Any product that may be evaluated in this article, or claim that may be made by its manufacturer, is not guaranteed or endorsed by the publisher.
