# Supplementary material for: Cyanobacteria as cell factories for the photosynthetic production of sucrose
Source: Front Microbiol. 2023 Feb 14;14:1126032. doi: 10.3389/fmicb.2023.1126032 (PMC9971976; doi:10.3389/fmicb.2023.1126032)
Supplement: Supplementary file 1 [file Data_Sheet_1.DOCX]

Supplementary Material

# Supplementary Figures and Tables


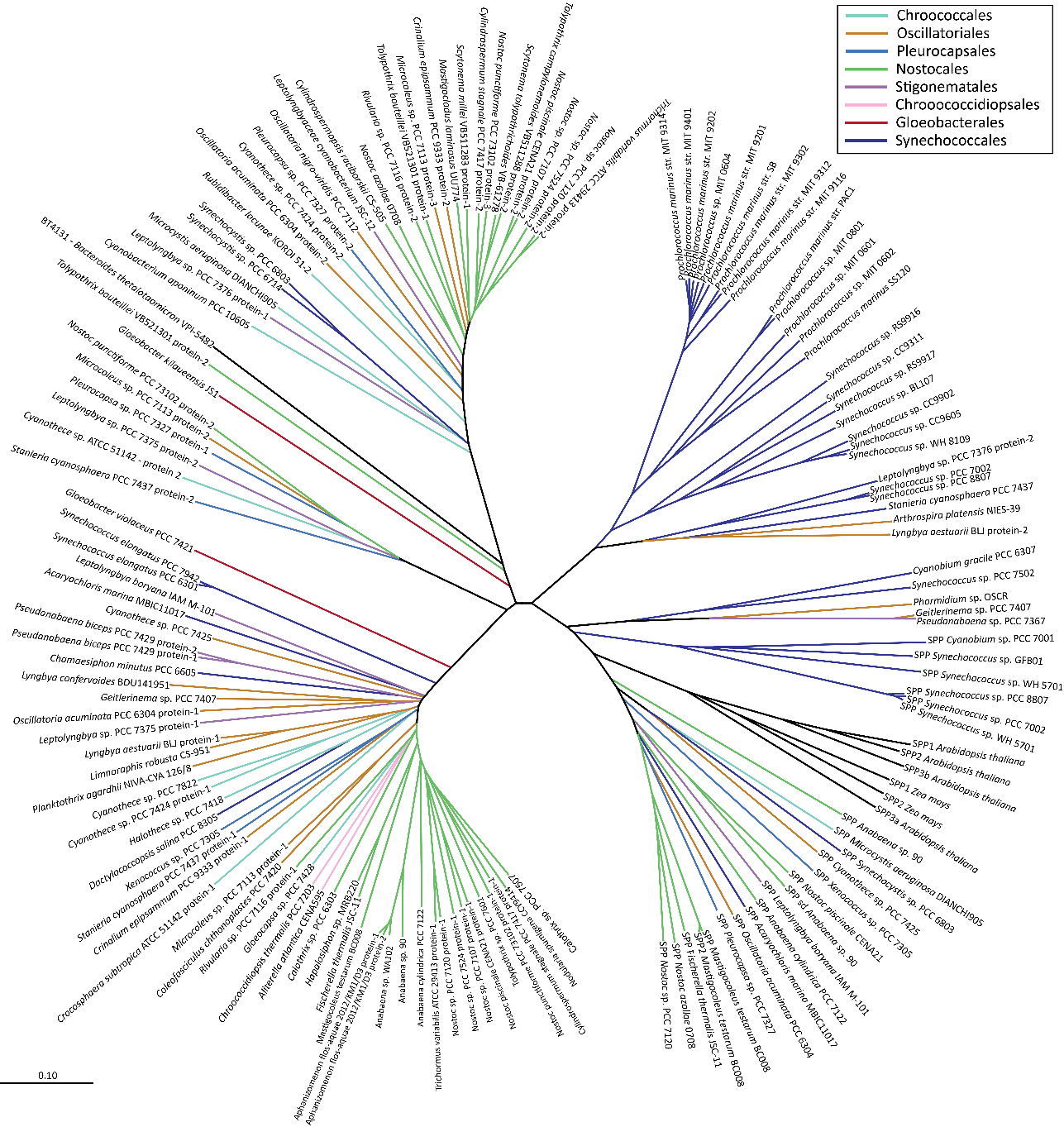


**Supplementary Figure S1.** Phylogenetic analysis of SPP-like proteins encoded by the genome of different cyanobacterial species. SPP proteins are indicated by SPP followed by the name of the strain, whereas SPP-like proteins are indicated only by the name of the strain. Unrooted Neighbor-joining phylogenetic trees were constructed after sequence alignment of the sequence of SPP proteins and SPP-like proteins using ClustalX with a BLOSSUM matrix and a bootstrap trial of 1000. The graphical representations of the trees were generated using FigTree. Sequences were obtained from the non-redundant protein databases of the National Center for Biotechnology Information by BLAST searches.

**Supplementary Table S1**. List of genes encoding proteins involved in the synthesis and degradation of sucrose in cyanobacteria.

| **Cyanobacterial strain** | **Morphology^a^** | **SPS (GTD)^b^** | **SPS (GTD-PHD)^c^** | **SPP** | **SuS** | **SPP-like** | **AMS** | **INV** |
| --- | --- | --- | --- | --- | --- | --- | --- | --- |
| *Acaryochloris marina* MBIC11017 | U | AM1_3195 |  | AM1_2884 | AM1_6048 | AM1_3755 | AM1_3468 | AM1_5358 |
| *Aliterella atlantica* CENA595 | U | UH38_00935 |  | UH38_15035 | UH38_12840 | UH38_13310 |  | UH38_24240, UH38_14840, UH38_12745 |
| *Anabaena cylindrica* PCC 7122 | FH | Anacy_0546 | Anacy_5147 | Anacy_4941 | Anacy_4232, Anacy_1886 | Anacy_4476 |  | Anacy_4286 |
| *Anabaena* sp. 90 | FH | ANA_C10718 | ANA_C12881 | ANA_C10157, ANA_C12945, ANA_C12946 | ANA_C11946 | ANA_C20735 |  | ANA_C12319 |
| *Anabaena* sp. WA102 | FH | AA650_24240 | AA650_11585 | AA650_21190 | AA650_01555 | AA650_10390 |  | AA650_03770 |
| *Aphanizomenon flos-aquae* 2012/KM1/D3 | FH | OA07_16000 | OA07_11135 | OA07_24745, OA07_15220 | OA07_04130 | OA07_12160, OA07_13485 |  | OA07_10250, OA07_20595 |
| *Arthrospira maxima* CS-328 | FNH |  |  |  | AmaxDRAFT_0499 |  |  |  |
| *Arthrospira platensis* C1 | FNH |  |  |  | SPLC1_S430370 |  |  |  |
| *Arthrospira platensis* NIES-39 | FNH |  |  |  | NIES39_O01860 | NIES39_H00600 |  |  |
| *Arthrospira* sp. PCC 8005 | FNH |  |  |  | ARTHRO_90007, ARTHRO_40478 |  |  |  |
| *Calothrix* sp. 336/3 | FH |  |  | IJ00_04815 |  |  |  | IJ00_05055 |
| *Calothrix* sp. PCC 6303 | FH | Cal6303_2893 | Cal6303_0199 | Cal6303_0610 | Cal6303_2136 | Cal6303_0069 |  | Cal6303_1384 |
| *Calothrix* sp. PCC 7507 | FH | Cal7507_6027, Cal7507_5994 |  | Cal7507_2549 | Cal7507_2215, Cal7507_5465 | Cal7507_6153 |  | Cal7507_6025 |
| *Chamaesiphon minutus* PCC 6605 | U | Cha6605_5458, Cha6605_0855 |  | Cha6605_0297 |  | Cha6605_0835 |  |  |
| *Chlorogloeopsis fritschii* PCC 6912 | FH | PCC6912_43130 |  | PCC6912_52440 | PCC6912_47490, PCC6912_13800 |  |  | PCC6912_37660, PCC6912_04940 |
| *Chroococcidiopsis thermalis* PCC 7203 | U | Chro_2260, Chro_2932 |  | Chro_0680 | Chro_5323 | Chro_1481 |  | Chro_0977, Chro_4248 |
| *Coleofasciculus chthonoplastes* PCC 7420 | FNH | MC7420_2965 |  | MC7420_3029 | MC7420_7584 | MC7420_6378 |  | MC7420_7297 |
| *Crinalium epipsammum* PCC 9333 | FNH | Cri9333_3742, Cri9333_4344 |  | Cri9333_1967 | Cri9333_2952 | Cri9333_1707, Cri9333_2099 |  | Cri9333_4050 |
| *Cyanobacterium aponinum* PCC 10605 | U |  |  |  |  | Cyan10605_3375 |  |  |
| *Cyanobium gracile* PCC 6307 | U |  | Cyagr_0980 | Cyagr_0979 |  | Cyagr_1586 | Cyagr_0977 |  |
| *Cyanobium* sp. PCC 7001 | U |  | CPCC7001_1239 | CPCC7001_1200 |  |  |  | CPCC7001_2022 |
| *Cyanothece* sp. ATCC 51142 | U |  |  | cce_1207 |  | cce_2437, cce_2301 |  |  |
| *Cyanothece* sp. PCC 7424 | U | PCC7424_3257, PCC7424_1935 |  | PCC7424_4476 | PCC7424_3776 | PCC7424_0088, PCC7424_3223 | PCC7424_3590 | PCC7424_2774 |
| *Cyanothece* sp. PCC 7425 | U | Cyan7425_1751, Cyan7425_2924 |  | Cyan7425_4592 | Cyan7425_1752, Cyan7425_3916 | Cyan7425_5151 |  |  |
| *Cyanothece* sp. PCC 7822 | U | Cyan7822_0598 |  | Cyan7822_2392 |  | Cyan7822_1598 |  | Cyan7822_5415 |
| *Cylindrospermopsis raciborskii* CS-505 | FH | CRC_02936 |  | CRC_00548 |  | CRC_03273 |  | CRC_00209 |
| *Cylindrospermum stagnale* PCC 7417 | FH | Cylst_1496 | Cylst_1866 | Cylst_0594 | Cylst_0728, Cylst_1832 | Cylst_5683, Cylst_3752 |  |  |
| *Dactylococcopsis salina* PCC 8305 | U | Dacsa_1438 |  | Dacsa_0649 | Dacsa_2708 | Dacsa_1127 |  |  |
| *Fischerella thermalis* JSC-11 | FH | FJSC11DRAFT_3760 |  | FJSC11DRAFT_2986 | FJSC11DRAFT_4368, FJSC11DRAFT_1280, FJSC11DRAFT_0905 | FJSC11DRAFT_4537 |  | FJSC11DRAFT_4015 |
| *Geitlerinema* sp. PCC 7407 | FNH | GEI7407_0058 |  | GEI7407_1610 | GEI7407_2715 | GEI7407_0606, GEI7407_2223 |  | GEI7407_3667 |
| *Gloeobacter kilaueensis* JS1 | U | GKIL_4105 |  | GKIL_4106 | GKIL_2834 | GKIL_4243 |  |  |
| *Gloeobacter violaceus* PCC 7421 | U | gll3840, gll3607 |  | gll3839 |  | gll2780 |  |  |
| *Gloeocapsa* sp. PCC 7428 | U |  |  | Glo7428_0776 | Glo7428_0408, Glo7428_4260 | Glo7428_2641 |  | Glo7428_3473 |
| *Halothece* sp. PCC 7418 | U | PCC7418_1738, PCC7418_2984 |  | PCC7418_1364 | PCC7418_2272 | PCC7418_3508 |  |  |
| *Hapalosiphon* sp. MRB220 | FH | AMR41_03555, AMR41_20985 |  | AMR41_05170, AMR41_20990 | AMR41_23745 | AMR41_17680 |  | AMR41_10465 |
| *Kamptonema formosum* PCC 6407 (*Hassallia byssoidea* VB512170) | FH |  |  | PI95_025755 | PI95_002775, PI95_009480 |  |  | PI95_015485 |
| *Leptolyngbya boryana* PCC 6306 | FNH | LBWT_5330, LBWT_24300 |  | LBWT_43300 | LBWT_4830, LBWT_25690 | LBWT_43350 |  | LBWT_28200, LBWT_11280 |
| *Leptolyngbya* sp. PCC 7375 | FNH |  |  |  |  | Lepto7375DRAFT_5438, Lepto7375DRAFT_2294 |  |  |
| *Leptolyngbya* sp. PCC 7376 | FNH |  |  |  |  | Lepto7376_1780, Lepto7376_0376 |  |  |
| *Leptolyngbyaceae cyanobacterium* JSC-12 | FNH | OsccyDRAFT_2725, OsccyDRAFT_2576 |  | OsccyDRAFT_4427 | OsccyDRAFT_3064 | OsccyDRAFT_4462 |  |  |
| *Limnoraphis robusta* CS-951 | FNH |  |  |  |  | WN50_20365 |  |  |
| *Lyngbya aestuarii* BL J | FNH |  |  |  |  | M595_4714, M595_3693 |  |  |
| *Lyngbya confervoides* BDU141951 | FNH |  |  |  |  | QQ91_18295 |  |  |
| *Lyngbya* sp. PCC 8106 | FNH |  |  |  |  |  |  |  |
| *Mastigocladus laminosus* UU774 | FH | BLD44_011515, BLD44_010800 |  | BLD44_010795, BLD44_004290 | BLD44_013670 | BLD44_017310 |  | BLD44_019245 |
| *Mastigocoleus testarum* BC008 | FH | BC008_31735, BC008_33390 |  | BC008_17795, BC008_44515 | BC008_17475 | BC008_38200 |  | BC008_42000, BC008_39810, BC008_40785 |
| *Microcoleus* sp. PCC 7113 | FNH | Mic7113_0204, Mic7113_1043, Mic7113_5916, Mic7113_5881 |  | Mic7113_1367 | Mic7113_3751, Mic7113_2737 | Mic7113_1091, Mic7113_2382, Mic7113_2016 |  |  |
| *Microcoleus vaginatus* FGP-2 | FNH | MicvaDRAFT_2906 |  | MicvaDRAFT_5193 | MicvaDRAFT_1609 |  |  |  |
| *Microcystis aeruginosa* DIANCHI905 | U | LRR78_19450 |  | LRR78_19460 | LRR78_19455 | LRR78_03260 |  |  |
| *Moorea producens* 3L | FNH | LYNGBM3L_56000, LYNGBM3L_59230 |  | LYNGBM3L_73410 | LYNGBM3L_31670, LYNGBM3L_74640 |  |  |  |
| *Neosynechococcus sphagnicola* sy1 | U |  |  | DO97_05200 | DO97_15625 |  |  |  |
| *Nodularia spumigena* CCY9414 | FH | NSP_50790 | NSP_8740 | NSP_21420 | NSP_48150, NSP_24720 | NSP_32550 |  | NSP_44650, NSP_44640 |
| *Nostoc azollae* 0708 | FH | Aazo_4553, Aazo_1310 |  | Aazo_2470 | Aazo_0215 | Aazo_0138 |  | Aazo_4707 |
| *Nostoc piscinale* CENA21 | FH |  |  | ACX27_02245 |  | ACX27_23780, ACX27_05375 |  | ACX27_23440, ACX27_23435 |
| *Nostoc punctiforme* PCC 73102 (ATCC 29133) | FH | Npun_R4579, Npun_F3065, Npun_R1558, Npun_F4709 |  | Npun_F3066, Npun_R3569 | Npun_F1876, Npun_F4951 | Npun_R5572, Npun_R5635, Npun_R6195 |  | Npun_F1611, Npun_F4643 |
| *Nostoc* sp. PCC 7107 | FH |  | Nos7107_1808 | Nos7107_4134 | Nos7107_1514, Nos7107_2802 | Nos7107_0074, Nos7107_3617 |  | Nos7107_3082 |
| *Nostoc* sp. PCC 7120 | FH | alr3370, all4376 |  | all0376 | all4985, all1059 | alr3714, alr3950 |  | alr0819, alr1521 |
| *Nostoc* sp. PCC 7524 | FH | Nos7524_1418 | Nos7524_3554 | Nos7524_0250 | Nos7524_5556, Nos7524_2894 | Nos7524_3252, Nos7524_0809 |  |  |
| *Oscillatoria acuminata* PCC 6304 | FNH | Oscil6304_2973, Oscil6304_2012, Oscil6304_0821, Oscil6304_3321 |  | Oscil6304_5556 | Oscil6304_3602 | Oscil6304_4363, Oscil6304_4200 |  |  |
| *Oscillatoria nigro-viridis* PCC 7112 | FNH | Osc7112_3724 |  | Osc7112_3132 | Osc7112_4334 | Osc7112_4431 |  |  |
| *Phormidium* sp. OSCR | FNH | HLUCCO16_05120 |  |  | HLUCCO16_00090 | HLUCCO16_05950 |  |  |
| *Planktothrix agardhii* NIVA-CYA 126/8 | FNH |  |  |  |  | A19Y_3564 |  |  |
| *Pleurocapsa* sp. PCC 7327 | U | Ple7327_3305, Ple7327_1732 |  | Ple7327_3241 | Ple7327_2003, Ple7327_0671 | Ple7327_0351, Ple7327_0375 |  |  |
| *Prochlorococcus marinus* str. GP2 | U | EU91_1876 |  |  |  |  |  |  |
| *Prochlorococcus marinus* str. MIT 9116 | U | EU93_0434 |  |  |  | EU93_1511 |  |  |
| *Prochlorococcus marinus* str. MIT 9201 | U | EU95_1566 |  |  |  | EU95_0257 |  |  |
| *Prochlorococcus marinus* str. MIT 9202 | U | P9202_382 |  |  |  | P9202_1597 |  | P9202_957 |
| *Prochlorococcus marinus* str. MIT 9302 | U | EU96_0777 |  |  |  | EU96_0488 |  |  |
| *Prochlorococcus marinus* str. MIT 9312 | U | PMT9312_1803 |  |  |  | PMT9312_0839 |  |  |
| *Prochlorococcus marinus* str. MIT 9314 | U | EU98_1511 |  |  |  | EU98_0557 |  |  |
| *Prochlorococcus marinus* str. MIT 9401 | U | EV01_1843 |  |  |  | EV01_1446 |  |  |
| *Prochlorococcus marinus* str. PAC1 | U |  | EV03_1131 |  |  | EV03_2054 |  |  |
| *Prochlorococcus marinus* str. SB | U | EV02_1242 |  |  |  | EV02_0189 |  |  |
| *Prochlorococcus* sp. MIT 0601 | U | EV05_0409 |  |  |  | EV05_0240 |  | EV05_0700 |
| *Prochlorococcus* sp. MIT 0602 | U | EV06_1559 |  |  |  | EV06_0904 |  | EV06_0432 |
| *Prochlorococcus* sp. MIT 0604 | U | EW14_2080 |  |  |  | EW14_0927 |  |  |
| *Prochlorococcus* sp. MIT 0701 | U |  | EV12_2408 |  |  |  |  |  |
| *Prochlorococcus* sp. MIT 0801 | U |  | EW15_2282 |  |  | EW15_0932 |  | EW15_0434 |
| *Pseudanabaena biceps* PCC 7429 | FNH |  |  | Pse7429DRAFT_3540 |  | Pse7429DRAFT_3930, Pse7429DRAFT_4687, |  |  |
| *Pseudanabaena* sp. PCC 7367 | FNH |  |  |  | Pse7367_0339 | Pse7367_0836 |  |  |
| *Richelia intracellularis* HH01 | FH |  |  | RINTHH_2980 |  |  |  | RINTHH_3860 |
| *Rivularia* sp. PCC 7116 | FH | Riv7116_1620 |  | Riv7116_6091 | Riv7116_6202, Riv7116_3519 | Riv7116_1336, Riv7116_1197 | Riv7116_3088 |  |
| *Rubidibacter lacunae* KORDI 51-2 | U | KR51_00033670 |  | KR51_00036500 | KR51_00015030 | KR51_00023650 |  |  |
| *Scytonema millei* VB511283 | FH | QH73_39920 |  | QH73_32320 | QH73_20650 | QH73_0023540 |  | QH73_19245 |
| *Scytonema tolypothrichoides* VB-61278 | FH | SD80_25365, SD80_08185 |  | SD80_030520 | SD80_004795, SD80_002085 | SD80_008830 |  | SD80_05170 |
| *Stanieria cyanosphaera* PCC 7437 | U | Sta7437_0828, Sta7437_0877 |  | Sta7437_2821 | Sta7437_0897 | Sta7437_1204, Sta7437_2824 |  | Sta7437_4400, Sta7437_0673 |
| *Synechococcus elongatus* PCC 6301 | U |  | syc0730_d |  |  | syc0956_c |  | syc1118_c |
| *Synechococcus elongatus* PCC 7942 | U |  | Synpcc7942_0808 |  |  | Synpcc7942_0566 |  | Synpcc7942_0397 |
| *Synechococcus* sp. BL107 | U |  | BL107_06684 |  |  | BL107_07029 |  |  |
| *Synechococcus* sp. CB0101 | U |  | CB0101_08175 |  |  |  |  | CB0101_14495 |
| *Synechococcus* sp. CC9311 | U |  | sync_2936 |  |  | sync_0362 |  | sync_0551 |
| *Synechococcus* sp. CC9605 | U |  | Syncc9605_2689 |  |  | Syncc9605_2605 |  |  |
| *Synechococcus* sp. CC9902 | U |  | Syncc9902_2315 |  |  | Syncc9902_2242 |  |  |
| *Synechococcus* sp. GFB01 | U |  | SYNGFB01_01875, SYNGFB01_01495 | SYNGFB01_01500 |  |  |  |  |
| *Synechococcus* sp. KORDI-49 | U | KR49_08880 |  |  |  |  |  | KR49_11485 |
| *Synechococcus* sp. KORDI-52 | U |  | KR52_02500 |  |  |  |  | KR52_00005 |
| *Synechococcus* sp. KORDI-100 | U | KR100_00785 |  |  |  |  |  | KR100_03925 |
| *Synechococcus* sp. PCC 6312 | U |  | Syn6312_2695 |  |  |  |  |  |
| *Synechococcus* sp. PCC 7002 | U |  | SYNPCC7002_A0888 | SYNPCC7002_A0887 |  | SYNPCC7002_A2023 | SYNPCC7002_A0885 |  |
| *Synechococcus* sp. PCC 7502 (RCC307) | U | SynRCC307_2529 |  |  |  | SynRCC307_0371 |  |  |
| *Synechococcus* sp. PCC 8807 | U |  | AWQ24_04545 | AWQ24_04540 |  | AWQ24_10365 | AWQ24_04530 |  |
| *Synechococcus* sp. RS9916 | U |  | RS9916_37502 |  |  | RS9916_28589 |  |  |
| *Synechococcus* sp. RS9917 | U |  | RS9917_05830 |  |  | RS9917_03523 |  | RS9917_02703 |
| *Synechococcus* sp. WH 5701 | U | WH5701_04810 |  | WH5701_04820, WH5701_04825 |  |  | WH5701_04830 |  |
| *Synechococcus* sp. WH 7803 | U |  | SynWH7803_2527 |  |  |  |  | SynWH7803_0536 |
| *Synechococcus* sp. WH 7805 | U |  | WH7805_09469 |  |  |  |  |  |
| *Synechococcus* sp. WH 8016 | U |  | Syn8016DRAFT_2816 |  |  |  |  | Syn8016DRAFT_2801 |
| *Synechococcus* sp. WH 8020 | U |  | WB44_11260 |  |  |  |  | WB44_13920 |
| *Synechococcus* sp. WH 8102 | U |  | SYNW2520 |  |  |  |  | SYNW1965 |
| *Synechococcus* sp. WH 8103 | U |  | SynWH8103_02908 |  |  |  |  | SynWH8103_02254 |
| *Synechococcus* sp. WH 8109 | U |  | Syncc8109_2757 |  |  | Syncc8109_2668 |  | Syncc8109_0527 |
| *Synechocystis* sp. PCC 6714 | U |  | D082_07330 | D082_32810 |  | D082_22870 |  |  |
| *Synechocystis* sp. PCC 6803 | U |  | sll0045 | slr0953 |  | sll1524 |  | sll0626 |
| *Thermosynechococcus elongatus* BP-1 | U |  | tlr0582 |  | tlr1047 |  |  |  |
| *Thermosynechococcus* sp. NK55a | U |  | NK55_11890 |  | NK55_01920 |  |  |  |
| *Tolypothrix bouteillei* VB521301 | FH | DA73_0400021370, DA73_0400007685 |  | DA73_0400029760 | DA73_0400004075, DA73_0400014025 | DA73_0400025145, DA73_0400022610, |  | DA73_0216535 |
| *Tolypothrix campylonemoides* VB511288 | FH | SD81_021240, SD81_032110 |  | SD81_034995 | SD81_000465, SD81_013585 | SD81_015670, SD81_018335 |  | SD81_023610 |
| *Tolypothrix* sp. PCC 7601 | FH | FDUTEX481_01133 | FDUTEX481_03174 | FDUTEX481_00686, FDUTEX481_07381 | FDUTEX481_05468, FDUTEX481_00440, FDUTEX481_00687 | FDUTEX481_08967 |  | FDUTEX481_09028, FDUTEX481_09026 |
| *Trichormus variabilis* ATCC 29413 | FH | Ava_3301, Ava_3411 |  | Ava_2821 | Ava_3753, Ava_2283 | Ava_3590, Ava_1752 |  | Ava_0609 |
| *Xenococcus* sp. PCC 7305 | U |  |  | Xen7305DRAFT_00023620 |  | Xen7305DRAFT_00028150 |  |  |

^a^FH, filamentous heterocyst-forming; FNH, filamentous nonheterocyst-forming; U, unicellular.

^b^SPS (GTD) refers to unidomainal SPS.

^c^SPS (GTD-PHD) refers to bidomainal SPS.

*Abbreviations used in this table: AMS, amylosucrase; GTD, glucosyltransferase domain; INV, invertase; PHD, phosphohydrolase domain; SPP, sucrose phosphate phosphatase; SPS, sucrose phosphate synthase; SuS, sucrose synthase.
